# Comprehensive Analyses Reveal Effects on Tumor Immune Infiltration and Immunotherapy Response of APOBEC Mutagenesis and Its Molecular Mechanisms in Esophageal Squamous Cell Carcinoma

**DOI:** 10.7150/ijbs.83824

**Published:** 2023-05-08

**Authors:** Jie Yang, Tao Xiang, Shihao Zhu, Yueqiong Lao, Yuqian Wang, Tianyuan Liu, Kai Li, Yuling Ma, Ce Zhong, Shaosen Zhang, Wen Tan, Dongxin Lin, Chen Wu

**Affiliations:** 1 Department of Etiology and Carcinogenesis, National Cancer Center/National Clinical Research Center for Cancer/Cancer Hospital, Chinese Academy of Medical Sciences and Peking Union Medical College, Beijing 100021, China; 2 Key Laboratory of Cancer Genomic Biology, Chinese Academy of Medical Sciences and Peking Union Medical College, Beijing 100021, China; 3 Collaborative Innovation Center for Cancer Personalized Medicine, Nanjing Medical University, Nanjing 211166, China; 4 Sun Yat-sen University Cancer Center, State Key Laboratory of Oncology in South China, Guangzhou 510060, China; 5 CAMS Oxford Institute, Chinese Academy of Medical Sciences, Beijing 100006, China

**Keywords:** esophageal squamous cell carcinoma, APOBEC signature, immune, APOBEC3A, immunotherapy

## Abstract

The apolipoprotein B mRNA editing enzyme catalytic polypeptide (APOBEC) mutagenesis is prevalent in esophageal squamous cell carcinoma (ESCC). However, the functional role of APOBEC mutagenesis has yet to be fully delineated. To address this, we collect matched multi-omics data of 169 ESCC patients and evaluate characteristics of immune infiltration using multiple bioinformatic approaches based on bulk and single-cell RNA sequencing (scRNA-seq) data and verified by functional assays. We find that APOBEC mutagenesis prolongs overall survival (OS) of ESCC patients. The reason for this outcome is probably due to high anti-tumor immune infiltration, immune checkpoints expression and immune related pathway enrichment, such as interferon (IFN) signaling, innate and adaptive immune system. The elevated AOBEC3A (A3A) activity paramountly contributes to the footprints of APOBEC mutagenesis and is first discovered to be transactivated by FOSL1. Mechanistically, upregulated *A3A* exacerbates cytosolic double-stranded DNA (dsDNA) accumulation, thus stimulating cGAS-STING pathway. Simultaneously, *A3A* is associated with immunotherapy response which is predicted by TIDE algorithm, validated in a clinical cohort and further confirmed in mouse models. These findings systematically elucidate the clinical relevance, immunological characteristics, prognostic value for immunotherapy and underlying mechanisms of APOBEC mutagenesis in ESCC, which demonstrate great potential in clinical utility to facilitate clinical decisions.

## Introduction

Somatic mutations accumulate throughout the process of tumor evolution. The cancer genome exhibits certain patterns of mutation, known as mutational signatures, reflecting the footprints of exogenous and endogenous mutational processes 1, 2. The APOBEC-related signatures, SBS2 and SBS13, are prevalent in ESCC according to our previous studies 3, 4. Moreover, it has been reported that SBS2 and SBS13 are present in the majority of ESCC samples and account for approximately 25% of the mutation burden 5, revealing that APOBEC mutagenesis is a crucial step in the evolutionary history of ESCC. APOBEC signatures are characterized by C-to-T or C-to-G changes at TCW motifs (where W refers to A or T). If the excessive mutations are not repaired, APOBEC-mediated mutational signatures will restrict tumor growth. Nevertheless, lower levels of mutations will promote tumor heterogeneity and cancer progression when the mutation load is not enough to restrict tumor 6.

Both SBS2 and SBS13 are contributed by APOBEC3 subfamily members. Petljak M and colleagues have provided evidence that there are causal links between APOBEC3 subfamily members and APOBEC-mediated signatures in bladder and breast cancer 7. The APOBEC3 enzyme subfamily consists of seven members, APOBEC3A-H (A3A, A3B, A3C, A3D, A3F and A3H), and their original main functions involve restriction of viral infection and genomic mobile elements 6. APOBEC3 enzymes participate in catalyzing cytidine deamination at TCW trinucleotide sequence context (where W refers to A or T) on single-stranded DNA (ssDNA) 8, 9. A3A and A3B are believed to be the main source contributing to APOBEC-mediated mutational signatures 10, 11, which exacerbate DNA replication stress-induced DNA damage and genome instability 12, 13.

Upon recognizing DNA damage-induced cellular DNA, cGAS, a cytosolic DNA sensor, participates in the innate and adaptive immune response pathways. Once activated, cGAS produces cGAMP, a second messenger that binds and activates STING, leading to the activation of type I IFN pathway, which facilitates the recruitment of T cells and the downstream anti-tumor immune response 14, 15. Concomitantly, DNA damage can trigger STAT1 signaling which further increases PD-L1 expression 16, 17. Recently, immunotherapy targeting PD-1/PD-L1 has exhibited promising effects, and it is becoming a standard line of treatment for ESCC 18, 19. However, the response of patients receiving immunotherapy is highly variable, and it is indispensable to identify effective biomarkers for predicting immunotherapy response. Accumulating evidences have corroborated that CD8+ T cells infiltration and the expression of immune checkpoints have been established as prerequisites for effective cancer immunotherapy 20, 21. Collectively, these findings drive us hypothesize that APOBEC mutagenesis has a potential impact on immunity and immunotherapy response. However, the direct consequences of APOBEC mutagenesis in ESCC tumor microenvironment have not been thoroughly addressed.

A mechanistic understanding of APOBEC mutagenesis will guide the use of immunotherapy. To this end, we investigate the genomic and transcriptomic profiles obtained from our previous two studies and the TCGA-Asian cohort 3, 4, 22. We have illustrated that APOBEC mutagenesis boosts CD8+ T cells infiltration and activates the immune response by deconvoluting bulk RNA sequencing (RNA-seq) data. Moreover, the analysis of scRNA-seq data further confirmed the observations. Mechanistically, A3A in tumor cells is the main mutator contributing to APOBEC mutagenesis in ESCC, and its overexpression is regulated by transcription factor (TF) FOS Like 1 (FOSL1). By stimulating cGAS-STING signaling, *A3A* elicits the infiltration of anti-tumor CD8+ T cells. In addition, *A3A* downregulation impairs the PD-L1 levels, and its expression level is found to be a robust predictor of the immunotherapy response which is validated in our mouse models and a clinical cohort. Taken together, our results provide insights into the impacts of APOBEC mutagenesis on ESCC, which leads to immotherapy benefits and might be useful for precision clinical cares of ESCC patients.

## Methods

### Data description

Somatic mutation, bulk RNA-seq and scRNA-seq data for 43 tumors from Cohort 1 were collected 4. 89 ESCC patients with somatic mutation, copy number variations, methylation profiles and bulk RNA expression profiles of tumors were obtained from Cohort 2 3. Bulk RNA-seq data of adjacent normal tissues from 145 patients with ESCC was also collected from these two cohorts. To eliminate the influence of genetic background, only 37 Asian patients with matched somatic mutation, copy number variations, methylation profiles and bulk RNA-seq data were recruited from the TCGA cohort 22. TGCA data were downloaded using the TCGAbiolinks R package (v1.15.1) 23. Details regarding sample collection, library construction, sequencing process and data analysis for these three cohorts were described elsewhere 3, 4, 22. The clinical characteristics and survival time of 169 patients with ESCC were shown in Table S1.

The expression matrix and clinical immunotherapy response information of IMvigor210 cohort were obtained via the R package IMvigor210CoreBiologies (v1.0.0).

### Mutational signature extraction

All exonic variants of 169 samples were used for mutational signatures analysis. Based on the trinucleotide sequence context, mutational signatures and the activities of each signature were extracted using SigProfiler. The inferred signatures were compared with Catalogue of Somatic Mutations in Cancer (COSMIC) reference signatures. The signature activity refers to the estimated number of mutations associated with each signature.

### APOBEC mutagenesis enrichment score (AMS) calculation

APOBEC mutagenesis strength was quantified using AMS by employing the following formula defined by Roberts et al. 24:



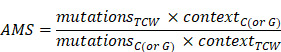



where 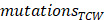 represented the counts of mutated C (and G) falling in a TCW (or WGA) motif, 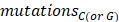 represented the total counts of mutated C (or G), 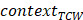 and 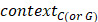 represented the total counts of TCW (or WGA) motifs and C (or G) occurring within 41-nucleotide region centered on the mutated bases.

RTCW and RTCA (R is a purine base and W represents A or T) events represented the number of mutated C (and G) falling in RTCW (or WGAY) and RTCA (or TGAY) motifs, respectively. YTCW and YTCA (Y is a pyrimidine base and W represents A or T) events represented the number of mutated C (and G) falling in YTCW (or WGAR) and YTCA (or TGAR) motifs, respectively.

### Survival analysis

The log-rank test was applied to conduct univariate survival analyses, and a multivariate Cox proportional hazards model was used in multivariate survival analyses with adjustments for clinical covariates such as age, gender, TNM stage, smoking and drinking status. The specific cut-offs we used to dichotomize AMS and *A3A* RNA levels for survival analysis were derived from the surv_cutoff function implemented in the survminer R package (v0.4.9).

### Mutation enrichment analysis

We performed mutation enrichment analysis as previously described 25 and interrogated the REACTOME database to identify pathways with significantly different number of mutations in ESCC. Fisher's exact test was applied, and the *P* value was adjusted by false discovery rate (FDR) correction by the Benjamin-Hochberg (BH) procedure. Pathways with *P* < 0.05 and FDR < 0.25 were considered significant. On the basis of these significant pathways, we assessed whether there was a significant different number of mutations between high AMS (HAMS) and low AMS (LAMS) tumors after FDR correction by BH procedure.

### Analysis of bulk RNA-seq data

Expression data (measured by TPM) of three cohorts were integrated. To remove the batch effects of different cohorts, we used the Combat function implemented in the R package sva (v3.42.0) 26. We filtered out the genes positively correlated with AMS to perform gene set enrichment analysis (GSEA) to assess the potential pathways enrichment associated with APOBEC mutagenesis using the R package clusterProfiler (v4.2.2). A *P* value obtained from GSEA less than 0.05 was considered to be enrichment significant. The activity of specific pathways extracted from gene set variation analysis (GSVA) was inferred by the GSVA R package (v1.42.0) 27. The immune cell fractions were estimated by the CIBERSORT algorithm based on the LM22 gene expression matrix (implemented in the R package immunedeconv v2.0.4) 28. The ESTIMATE immune score, ESTIMATEScore, StromalScore and TumorPurity were generated by the ESTIMATE algorithm 29. Another way to predict immune infiltration was via the R package TMEscore (v0.1.4) 30. The CYT score was calculated as the geometrical mean of* PRF1* and *GZMA* 31. The random forest algorithm was employed to quantify the importance of APOBEC3 members to TCW mutations and AMS using the R package randomForest (v4.7-1.1). The TIDE score was used to measure the efficacy of immune checkpoint blockade (ICB), which was generated from the TIDE website (http://tide.dfci.harvard.edu/) based on bulk RNA expression profiles 32.

### Processing of scRNA-seq data from 43 patients with ESCC

ScRNA-seq data was retrieved from the Gene Expression Omnibus (GEO; https://www.ncbi.nlm.nih.gov/geo/) database (GSE160269). In brief, only cells with 300-8000 genes detected, no more than 20000 total counts and 10% of mitochondrial gene counts were used for the further data analysis using the standard Scanpy (version 1.8.2) workflow 33. Gene counts were normalized and highly variable genes were selected with 0.0125-3 mean expression and more than 0.5 dispersion of genes. The expression of key stress genes was regressed out to remove the influence of stress markers (Table S2). We performed principal component analysis (PCA). To remove the batch effects, the BBKNN algorithm was used with patients as the batch term 34. Then cell clusters were identified using Leiden graph-clustering method and cell type annotation was used as previously published 35.

To examine the enrichment of each cell type across different groups, we used the following formula as previously mentioned 36:


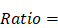


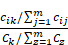


where 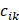 represented the counts of a cell type 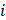 in one group (named group 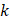), 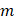 represented total counts of group, and 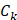 represented the total counts of cells in group 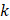. One cell type was assumed to be enriched in a group when 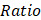 > 1.

GSEA analysis for epithelial and T cells was performed using the top 400 genes positively correlated with AMS. Genes used for antigen presentation score calculation in epithelial cells were identical to those used in our previous study 4. For CD4+ T cells, correlations between the reference gene *IL2RA* and other differentially expressed genes across all CD4+ T cell subtypes were calculated, and the top 30 correlated genes were used to define the regulation score. The *HAVCR2* gene was taken as the reference gene in CD8+ T cells, and the same method was used to define exhuastion score (Table S3). Cells from each subtype of CD4+ and CD8+ T cells were randomly downsampled to 1000 cells for trajectory analysis to estimate the developmental pseudotime using Monocle2 (version 2.22.0) with default parameters 37.

### Cell culture and treatment

Human ESCC cell lines (KYSE30 and KYSE510) were gifted by Dr. Y. Shimada of Hyogo College of Medicine, Japan. They were all authenticated by DNA fingerprinting analysis and tested free of mycoplasma infection. Mouse ESCC cell line (mEC25) was gifted by Dr. Li Fu of Shenzhen University International Cancer Center 38. Human ESCC cell lines and mEC25 cells were respectively maintained in RPMI 1640 and DMEM medium supplemented with 10% fetal bovine serum at 37°C in a humidified incubator with 5% CO2. Cis-dichlorodiammineplatinum (CDDP) (MCE, HY-17394) was dissolved in dimethylsulfoxide (DMSO), and cells were treated with 2 μM CDDP or DMSO for 12 hours.

### Establishment of cell lines with altered *A3A* expression

The *A3A* (NM_145699) sequence was cloned into the GV492 vector (Ubi-MCS-3FLAG-CBh-gcGFP-IRES-puromycin) for establishing cell lines with ectopic overexpression of *A3A*. The *A3A* knockout cell lines were generated by the CRISPR/Cas9 system. Cas9 and single-guide RNA sequences targeting the genomic *A3A* sequence were designed using the CRISPR design tool and cloned into the GV708 plasmid (U6-sgRNA-EF1a-Cas9-FLAG-CMV-EGFP-P2A-puro). The negative control sequence was CGCTTCCGCGGCCCGTTCAA, and the knockout target sequences were GACCTACCTGTGCTACGAAG and ATGGAAGCCAGCCCAGCATC. These packed lentiviruses were purchased from Genechem (Shanghai). To establish stable *A3A* overexpression or knockout cell lines, KYSE30, KYSE510 and mEC25 cells were infected with the lentivirus and cultured in complete medium for 48 hours, followed by selecting with 2.5 μM puromycin for one week. The stable overexpression and knockout cell lines were verified by Western blotting assays.

### Quantification of cytosolic DNA

Cells in 10-cm dishes were washed with PBS, harvested and subjected to nuclear and cytosolic fractionation using the MinuteTM Plasma Membrane Protein Isolation and Cell Fractionation Kit (Invent). Nuclear and cytosolic DNA was extracted by HiPure Universal DNA Kit (Magen). The dsDNA was quantified using Qubit 4.0 (Invitrogen) with Qubit dsDNA HS Reagent (Vazyme, EQ121-02-AA). The relative cytosolic DNA level was calculated as the cytosolic-to-nuclear DNA ratio.

### Immunofluorescence analysis

Cells were seeded on the tissue culture-treated coverslips (Solarbio) in a 24-well plate. When reached approximately 50% confluency, cells were washed with cold PBS and fixed with cold methanol at -20°C for 10 minutes. After being washed three times with PBS, the cells were blocked with 3% bovine serum albumin in PBS for 1 hour. The coverslips were then probed with Pico488 dsDNA quantification reagent for 1 hour and stained with γH2AX antibody (phospho S139, ab26350, Abcam) and cGAS antibody (#79978, Cell Signaling Technology) overnight. After being washed three times with PBS, the cells were incubated with the secondary antibody Alexa Fluor 555 (ThermoFisher) or Alexa Fluor 488 (ThermoFisher) for 1 hour. Coverslip was mounted with mounting medium containing DAPI (ZSGB-BIO), and the images were captured by confocal microscopy (Perkin Elmer).

### Western blot assays

Cells were lysed with RIPA lysis buffer (Solarbio, R0020) containing PMSF (Solarbio, P0100), phosphatase inhibitor cocktail I (MCE, HY-K0021) and phosphatase inhibitor cocktail II (MCE, HYK0022). Total protein levels in cell lysates were determined by the BCA kit (Thermo Fisher Scientific). Lysates containing 10-20 μg of protein were subjected to SDS-PAGE separation and transferred to the PVDF membrane (Millipore) for determination. The antibody used for A3A (PA5-99584) was from Thermo Fisher. Vinculin (ab219649), STAT1 (ab234400), γH2AX (phospho S139, ab26350) antibodies were from Abcam, while antibodies against cGAS (#79978), STING (#13647), phospho-STING (p-STING) (Ser366; #50907), IRF3 (#11904), phospho-IRF3 (p-IRF3) (Ser396; #29047), TBK1 (#3504), phospho-TBK1 (p-TBK1) (Ser172; #5483), and phospho-STAT1 (p-STAT1) (Tyr701; #7649) were from Cell Signaling Technology. Antibodies against for PD-L1 (66248-1-Ig) was from Proteintech. All the antibodies were diluted in Universal Antibody Diluent (NCM Biotech, WB500D). The membrane was incubated overnight at 4°C with primary antibody and 2 hours with secondary antibody. The signal was detected with a SuperSignalTM West Pico/Femto Chemiluminescent Substrate kit (Thermo Fisher, 34580) through the Amersham Imager 600.

### Quantitative real-time PCR

Total RNA was extracted from cells using the RNA-Quick Purification Kit (ES Science, RN001) according to the manufacturer's instructions. PrimeScript RT reagent kit and SYBR Premix Ex Taq II kit (Takara) were used for the detection of mRNA expression through an ABI 7900HT Real-Time PCR system using the primers shown in Table S4. Each sample was detected in triplicate, and individual RNA levels were determined relative to *GAPDH* RNA levels.

### Immunohistochemistry (IHC)

Formalin-fixed paraffin-embedded (FFPE) tissue sections were deparaffinized with Histo-Clear II twice and rehydrated with gradient ethanol, followed by inactivating of endogenous peroxidase and retrieval antigen. For IHC staining, the sections were stained by antibodies against A3A (1:300, PA5-99584, Thermo Fisher), and FOSL1 (1:50, sc-28310, Santa Cruz) at 4°C overnight and then detected with the ABC Kit (Pierce). The labeling score of intensity was estimated as negative (0), weak (1), moderate (2) and strong (3). The extent of staining, defined as the percentage of positive stained cells, was scored as 1 (≤ 10%), 2 (11%-50%), 3 (51%-80%) and 4 (> 80%). The total immune reactive score was obtained by multiplying the staining score of intensity and that of extent, ranking from 0 to 12.

### Multiplex immunofluorescent assays

The sections were deparaffinized, and rehydrated as described for the IHC procedure. Opal multiplex staining was performed according to the Opal 5-Color Manual IHC Kit (PANOVUE). The sections were incubated by antibodies against A3A (1:300, PA5-99584, Thermo Fisher), CD8 (1:800, ab93278, Abcam), Granzyme B (1:300, #17215, CST), PD-L1 (1:400, ab205921, Abcam), and FOSL1 (1:50, sc-28310, Santa Cruz) at 4°C overnight. Opal 520 corresponding to the A3A antibody, Opal 570 to Granzyme B (or PD-L1) antibodies and Opal 650 to CD8 (or FOSL1) antibodies were used to generate different immunofluorescent signals. Slides were counterstained with DAPI for nuclei visualization, and subsequently coverslipped using a VectaShield Hardset mounting media. The slides were imaged using Vectra Polaris Automated Quantitative Pathology Imaging System (Perkin Elmer). We used inForm software (Perkin Elmer) to unmix and remove autofluorescence and analyze the multispectral images.

### RNA interference of gene expression

KYSE30 and KYSE510 cells were plated at 30%-40% confluency and incubated overnight. Transfection of each small interfering RNA (siRNA) (60 nmol/L) into cells was performed with jetPRIME® (Polyplus) following the manufacturer's instructions. The siRNAs targeting for *FOSL1*, *NFKB1* and *VEZF1* (Table S4) were purchased from GenePharma.

### Construction of reporter plasmid and dual luciferase reporter assay

DNA containing the *A3A* promoter region produced by PCR amplification was cloned into the GV238 vector (MCS-firefly_Luciferase). The wild-type (WT) plasmid with the FOSL1 binding motif and the mutant (Mut) plasmid containing the FOSL1 binding motif deletion variant were designed by Genechem (Shanghai). KYSE510 cells were seeded in 48-well plates and infected with these plasmids using jetPRIME® (Polyplus). One day after silencing *FOSL1* by siRNA, KYSE510 cells were transfected with the WT plasmid. When cells grew to 80-90% confluency, luciferase reporter assays were performed according to the manufacturer's instructions (E1910, Promega).

### Chromatin immunoprecipitation (ChIP)

ChIP experiments were performed using the SimpleChIP Enzymatic Chromatin IP kit (#9003, CST) according to the manufacturer's protocol. Cells in two 15-cm dishes were cross-linked with formaldehyde solution (#MKCM8973, Sigma) for 10 minutes at room temperature followed by quenching with glycine. The cells were then lysed and the chromatin was fragmented by the focused-ultrasonicator (S220, Covaris). Chromatin complexes were immunoprecipitated with FOSL1 antibody (#5281, CST). The precipitated DNA samples were quantified by qPCR. Data are expressed as the percentage of input DNA and normalized as the expression fold change of IgG. The primer sequences used for ChIP-quantitative real-time PCR (ChIP-qPCR) are listed in Table S4.

### Mouse tumor experiments

Ten 6-week-old C57BL/6 mice were randomly divided into two groups. The mouse ESCC cells (6 × 10^6^), named mEC25, with or without *A3A* overexpression suspending in 200 μL of PBS containing 100 μL growth factor-reduced Matrigel (354230, Corning) were subcutaneously injected into the right hind legs of mice. For the anti-PD-1 monotherapy, twenty mice inoculated mEC25 cells with or without *A3A* overexpression were randomly divided into 4 groups (N = 5 per group) and the antibodies were administered 11 days post inoculation. 200 μg/mouse of anti-PD-1 antibody (BE0146, BioXCell) and anti-IgG isotype control antibody (BE0089, BioXCell) were intra-tumoral injected for a total of 5 doses every 2 days. Tumor volume was assessed by manual caliper measurements every 2 days. Tumors were collected 21 days after transplantation and their volumes were determined by 0.5 × length × width^2^. The animal experiments and procedures were approved by the Institutional Animal Care and Use Committee of the Chinese Academy of Medical Sciences.

### Statistical analysis

Two-sided Wilcoxon rank sum tests were conducted to determine whether there were statistically significant differences between two abnormal distributions. Spearman correlation was employed to examine the correlation between two continuous variables. For functional assays results, the comparison of measurements between two groups was performed by Student's t-test and all data was presented as the mean ± SEM. A *P* value of less than 0.05 was used as the criterion for statistical significance. Statistical analysis was performed using R 4.1.3, python v3.8.12 or GraphPad Prism v7.04.

## Results

### The APOBEC mutagenesis is prevalent in ESCC

**Figure 1:**
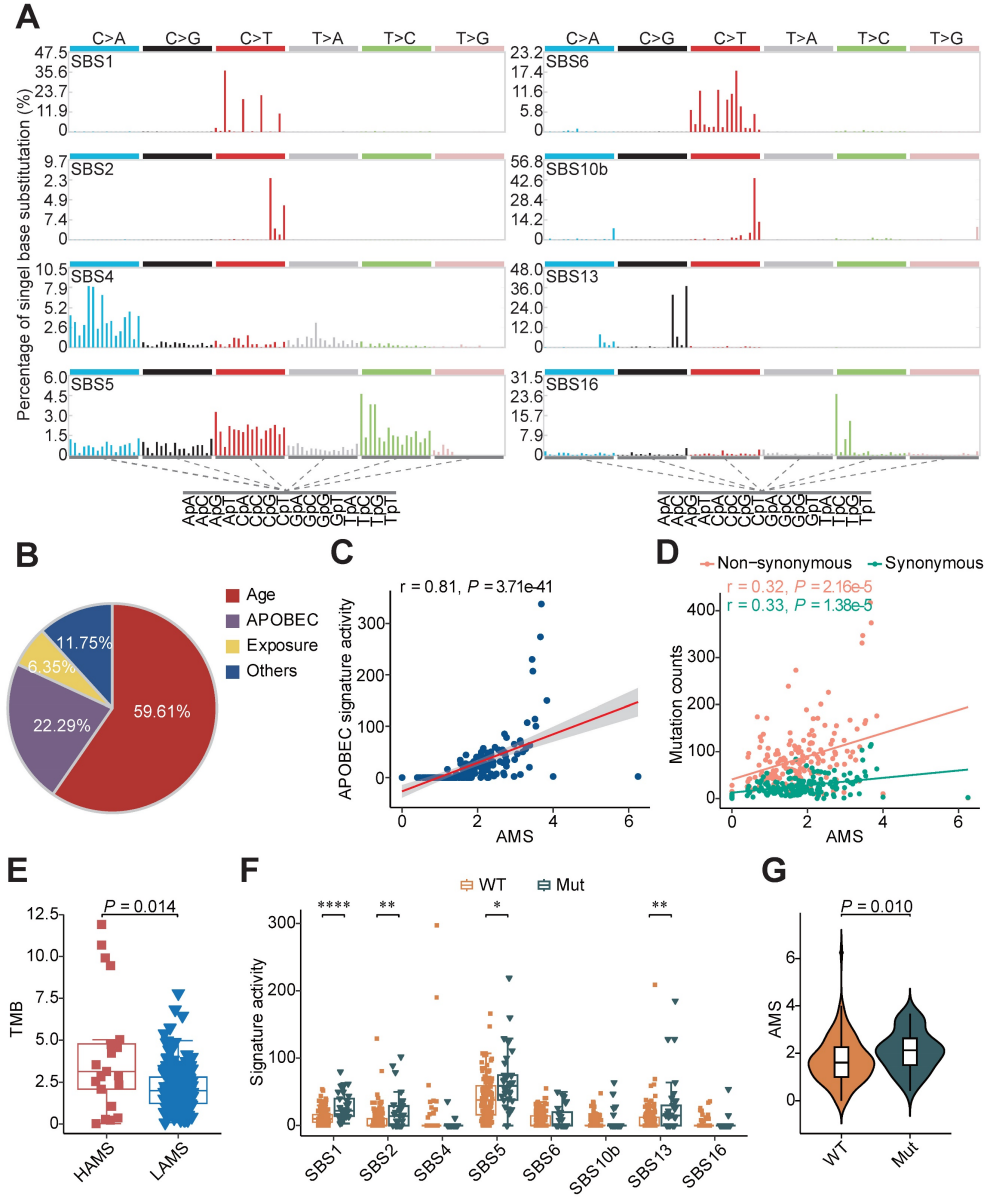
** Mutational signatures analysis in ESCC.** (**A**) Eight distinct mutational signatures were identified in 169 ESCC tumors. The x-axis denoted the 96 types of trinucleotide context sequence, and the y-axis denoted the percentage of the detected signature. (**B**) Pie charts showing the percentage of mutations assigned to each signature in ESCC described in **A**. Age: SBS1 and SBS5; APOBEC: SBS2 and SBS13; Exposure: smoking-related SBS4 and drinking-related SBS16; Others: SBS6 and SBS10b. (**C**) Scatter plot showing positive correlation between AMS and APOBEC signature activity. (**D**) AMS was positively correlated with both non-synonymous (pink) and synonymous (green) mutation counts in ESCC. (**E**) Boxplot showing TMB comparison between different AMS groups. TMB was measured by the counts of non-synonymous SNVs and indels per megabase. (**F**) Boxplots showing mutational signature activitity described in **A** across patients with or without somatic mutations in DDR genes. WT: wild type; Mut: mutant. The *P* value of Wilcoxon rank sum test represented the significance. * indicating *P <* 0.05, ** indicating *P <* 0.01, *** indicating *P <* 0.001, **** indicating *P <* 0.0001. (**G**) Violin plot showing AMS difference across patients with at least one or without somatic mutations in DDR genes. Boxplots in (**E-F**) displayed the median (central line), the 25-75% interquartile range (IQR) (box limits), the ±1.5 times IQR (Tukey whiskers), respectively.

A total of 169 patients with ESCC from three published cohorts were included in our study 3, 4, 22. To dissect potential genomic mutational processes in ESCC, we analyzed the distribution of six types of single nucleotide variations (SNVs) (C>A, C>G, C>T, T>A, T>C and T>G) and observed that C>T transitions were the most common type (Figure S1A). Further analysis of the trinucleotide sequence contexts showed enrichment of 5'-T [C>T] W-3' and 5'-T [C>G] W-3' (where W was A or T), which was highly suggestive of APOBEC mutagenesis in ESCC (Figure S1A). De-novo mutational signature analysis was performed using SigProfiler and eight COSMIC reference signatures were decomposed 1, including age-related SBS1 and SBS5, APOBEC-related SBS2 and SBS13, smoking-related SBS4, mismatch repair deficiency-related SBS6, polymerase epsilon exonuclease domain mutation-related SBS10b and drinking-related SBS16 (Figure 1A). APOBEC-induced signatures contributed approximately 22.29% of the mutation burden, which was similar to the result of a previous report 5. Besides the age associated signatures SBS1 and SBS5, APOBEC-induced signatures were dominant in ESCC sequence feature (Figure 1B).

To characterize the strength of mutagenesis at the TCW motif, we calculated the AMS as Roberts et al. defined 24. We found that the AMS was highly positively correlated with APOBEC signature activity (Figure 1C) and the proportion of mutations at TCW motifs was significantly elevated in the HAMS group compared with the LAMS group (Figure S1B). Given that APOBEC mutagenesis is generated by cytosine deamination resulting in C>T or C>G mutations, we analyzed the association between AMS and mutation burden, and found that AMS was significantly correlated with the numbers of both non-synonymous and synonymous mutations (Figure 1D). In addition, the tumor mutation burden (TMB) elevated as the AMS increased, which suggested that higher AMS might be associated with higher immunogenicity (Figure 1E).

APOBEC-related mutations were retained in the cancer genome depending not only on the expression of APOBEC proteins, but also the lesion not properly repaired. To investigate the effects of DNA damage repair (DDR) system on APOBEC mutagenesis, we examined the mutational profiles of DDR genes in ESCC. More than 21% of ESCC patients had somatic alterations in DDR genes (Figure S1C). Patients with one or more somatic mutations in these genes displayed higher age- and APOBEC-related signature activity and higher AMS than patients without (Figures 1F and 1G). The results showed that APOBEC mutagenesis interacted or had synergy with somatic mutations in DDR genes.

### Higher AMS is correlated with favorable prognosis in ESCC

**Figure 2:**
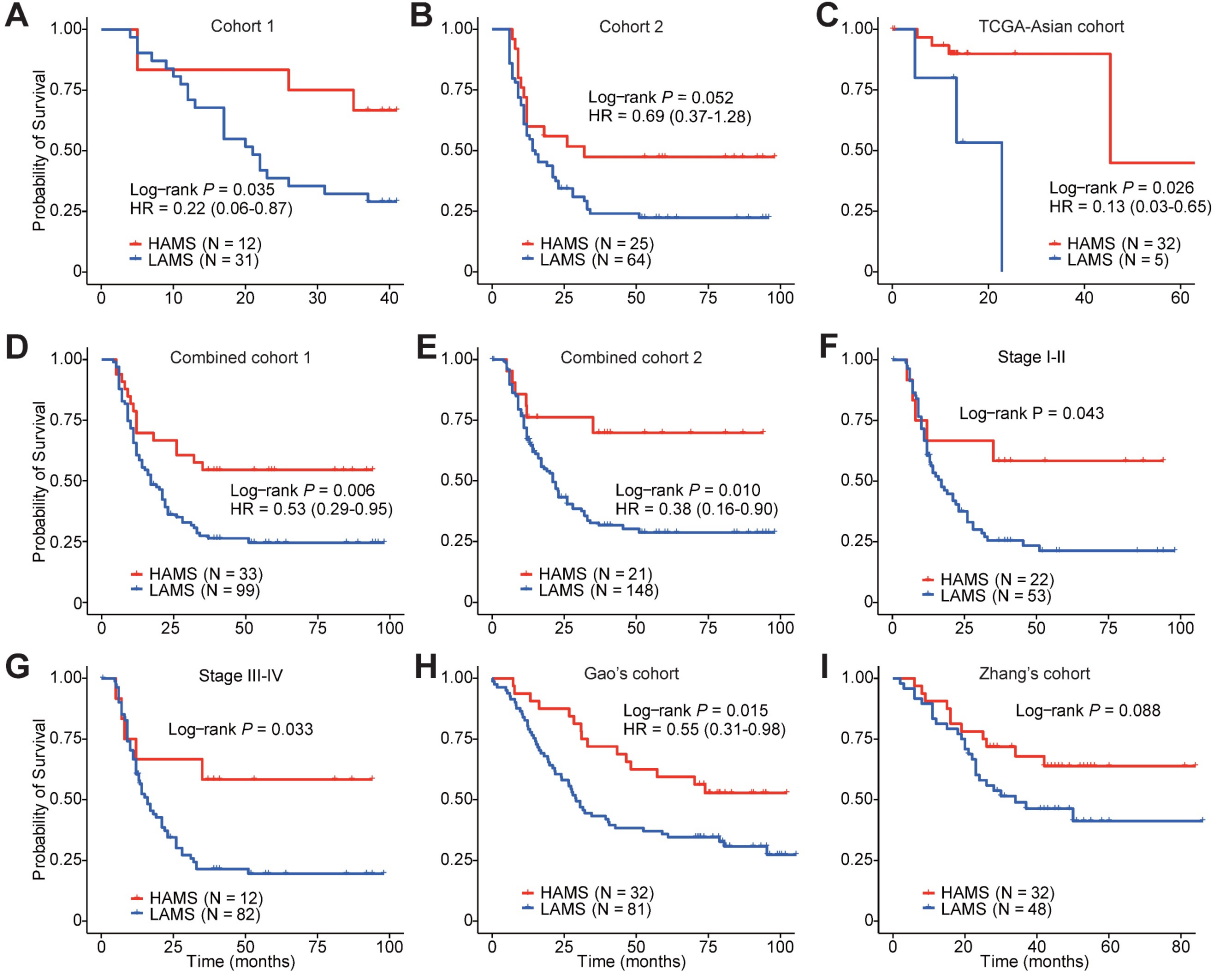
** Survival-based clinical relevance of AMS in ESCC.** (**A-E**) The Kaplan-Meier survival curves and multivariate analyses showing the cumulative risk of AMS in three independent cohorts (Cohort 1, Cohort 2 and TCGA-Asian cohort), combined cohort 1 combining two our own cohorts (Cohort 1 and Cohort 2) and combined cohort 2 combing these three cohorts. (**F-G**) Subgroup survival analysis in ESCC patients of stage I-II and stage III-IV. (**H-I**) Associations between AMS and OS were further validated in two independent cohorts. *P* values were derived from log-rank test. HRs and 95% CI derived from multivariate Cox proportional hazard models adjusting age, gender, clinical stage, smoking and drinking status were presented.

To explore the clinical relevance of AMS, we evaluated the predictive value of AMS for OS. Survival analyses were performed in three cohorts and two combined cohorts using the optimal cut-off with the minimum log-rank *P* value by testing a series of values of that AMS with fixed increments. Individuals with higher AMS had significantly longer survival time in three separate cohorts (*P*_log-rank_ = 0.035, *P*_log-rank_ = 0.052 and *P*_log-rank_ = 0.026, respectively. Figures 2A-C) or the two combined cohorts (*P*_log-rank_ = 0.006 and *P*_log-rank_ = 0.010, Figures 2D and 2E). Cox proportional hazards model analyses showed that after adjusting for potentially confounding factors such as age, gender, TNM stage, smoking and drinking status, higher AMS was still significantly associated with improved OS time, with the adjusted hazard ratios (HRs) of 0.22 (95% confidence interval (CI) = 0.06-0.87), 0.69 (95% CI = 0.37-1.28) and 0.13 (95% CI = 0.03-0.65) for the three independent cohorts (Figures 2A-C), and 0.53 (95% CI = 0.29-0.95) and 0.38 (0.16-0.90) for the two combined cohorts respectively (Figures 2D and 2E).

Subgroup survival analyses also showed differential OS time between the two AMS groups in both early- and late-stage ESCC patients (*P*_log-rank_ = 0.043 and *P*_log-rank_ = 0.033, Figures 2F and 2G). In a follow-up validation study using two independent public datasets, the survival analyses also confirmed the conclusion, although Zhang's cohort just reach a borderline significance (*P*_log-rank_ = 0.015 and *P*_log-rank_ = 0.088, Figures 2H and 2I) 39, 40. Together, these observations pinpointed that AMS was a protective prognostic biomarker for ESCC patients' survival.

### APOBEC mutagenesis promotes the anti-tumor immune response

**Figure 3:**
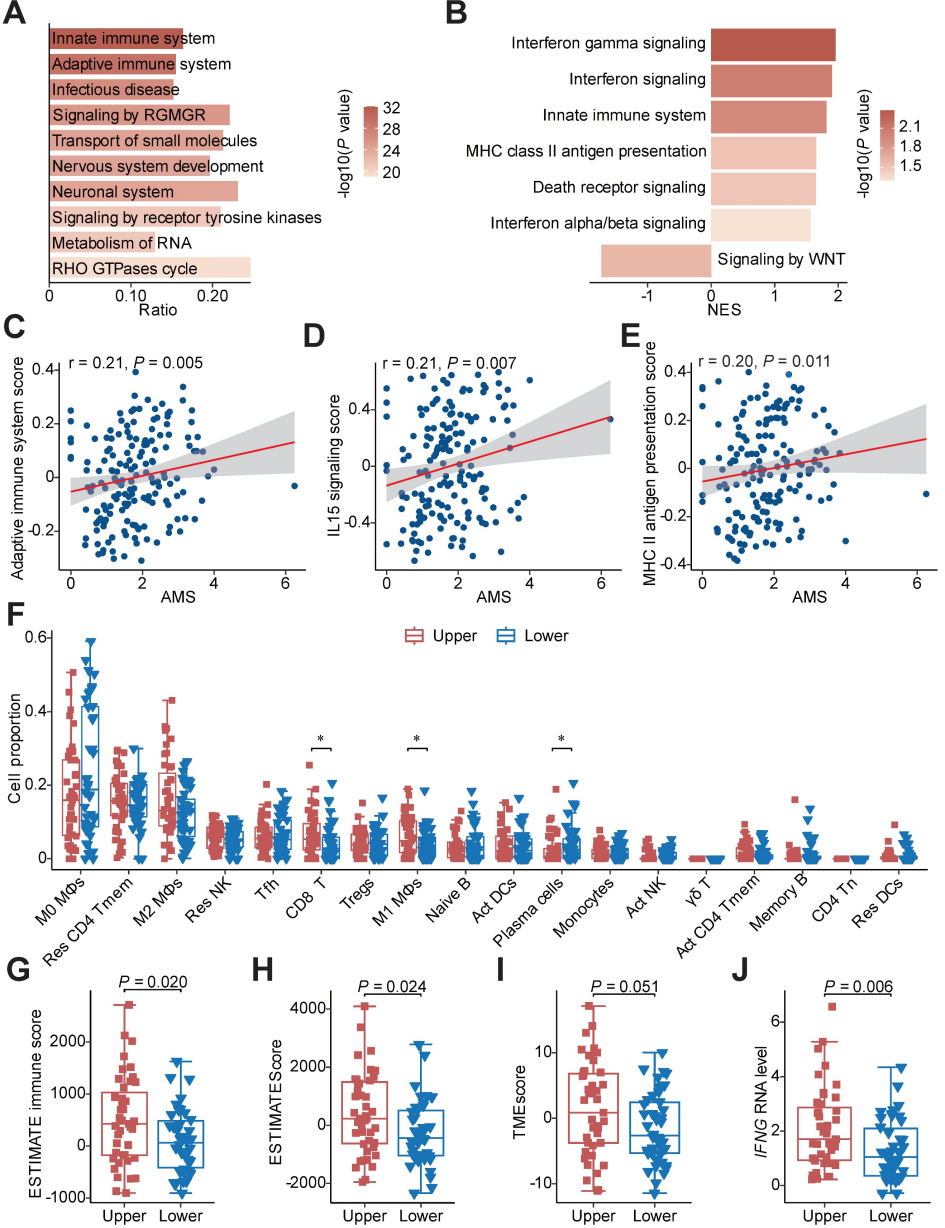
** Functional pathways annotation and immune infiltration comparison between HAMS and LAMS groups.** (**A**) The top 10 significant pathways by mutation enrichment analysis between HAMS and LAMS patients. RGMGR: RHO GTPases, Miro GTPases and RHOBTB3. (**B**) GSEA results showing significantly enriched pathways using genes positively correlated with AMS. (**C-E**) Spearman correlations between AMS and activity of specific immune related pathways measured by GSVA. (**F**) The proportion comparison of immune cells estimated by CIBERSORT between patients with upper and lower quantile of AMS. Mφ: macrophages; Res: resting; Tmem: memory T cells; Tfh: follicular helper T cells; Tregs: regulatory T cells; Act: activated; γδ T: gamma delta T cells; Tn: naive T cells; DCs: dendritic cells. The *P* value of Wilcoxon rank sum test represented the significance. * indicating *P <* 0.05. (**G-H**) Boxplots comparing ESTIMATE immune score and ESTIMATEScore estimated by ESTIMATE algorithm. The latter is a comprehensive assessment of immune and stromal score. (**I-J**) TMEscore (**I**) and *IFNG* mRNA expression (**J**) comparison between different AMS groups. Boxplots in (**F-J**) displayed the median (central line), the 25-75% IQR (box limits), the ±1.5 times IQR (Tukey whiskers), respectively.

To investigate the mechanism of favourable survival, we conducted mutation enrichment analysis using whole genome sequencing (WGS) or whole exome sequencing (WES) data and GSEA using bulk RNA-seq data to identify enriched pathways in HAMS samples as previously described 25, 41. The analysis at the mutation level showed a significant enrichment of mutations in pathways involved in the innate immune system (odds ratio = 3.20; FDR = 1.06e-29) and adaptive immune system (odds ratio = 3.70; FDR = 1.51e-27) in HAMS tumors, implying that APOBEC mutagenesis was vital in directing mutations in innate and adaptive immune-related genes (Figure 3A). As expected, the results of GSEA using bulk RNA-seq data also demonstrated a significant overexpression of innate immune system and MHC class II antigen presentation pathway as AMS elevated (Figure 3B). Interestingly, the type I and type II IFN signaling were significantly enhanced while WNT signaling was impaired during the APOBEC mutagenesis process. Several important genes involved in these pathways positively correlated with AMS were presented in Figures S2A-C. Type I IFN signaling was induced as a downstream key effector of the cGAS-STING pathway for promoting antigen presentation and immune activation 42, 43. The effects of double-stranded breaks (DSBs) on type II IFN signaling activation have been highlighted previously 44. Downregulation of the WNT pathway mainly exerted its tumor-suppression effect 45. Our data demonstrated the anti-tumor function of APOBEC mutagenesis.

We next quantified the aberrant activities of Reactome pathways measured by GSVA scores and consistently discovered that the activities of several immune activation-associated pathways, such as adaptive immune system, IL15 signaling and MHC class II antigen presentation, were moderately elevated in tumors with higher AMS (Figures 3C-E) 27. To further characterize the difference between patients with high and low AMS, we defined upper and lower quantile groups using the upper and lower quantiles of AMS. Besides all the pathways previously mentioned, TCR signaling, CD28 co-stimulation and PD-1 signaling were conceivably significantly stimulated in upper quantile group than lower quantile group (Figure S2D).

Considering the relationship between APOBEC mutagenesis and immune activity, we estimated the absolute fraction of immune cell types in ESCC using bulk RNA-seq data and found that individuals with higher AMS had significantly increased fractions of CD8+ T cells and M1 macrophages and decreased proportions of plasma cells (Figure 3F). Several indexes indicating immune infiltration status including ESTIMATE immune score, ESTIMATEScore, TMEscore and StromalScore increased (Figures 3G-I and S2E), while TumorPurity decreased in the upper quantile group (Figure S2F) using the ESTIMATE and TMEscore algorithms to evaluate the tumor microenvironment score. In view of IFNγ is an important immune regulation factor, we then compared the difference between the two groups and observed augmented *IFNG* mRNA expression levels in the upper quantile group (Figure 3J). Subsequently, we examined whether APOBEC mutagenesis had significant associations with cancer immunotherapy in ESCC. In the process of APOBEC mutagenesis, the increased expression of important immune checkpoint molecules, including *PDCD1*, *TIGIT*, *HAVCR2*,* LAG3* and* IDO1*, and the elevated CYT score, which serves as cytotoxic effects and anti-tumor response, were highly suggestive of better immunotherapy response (Figures S2G and S2H).

**Figure 4:**
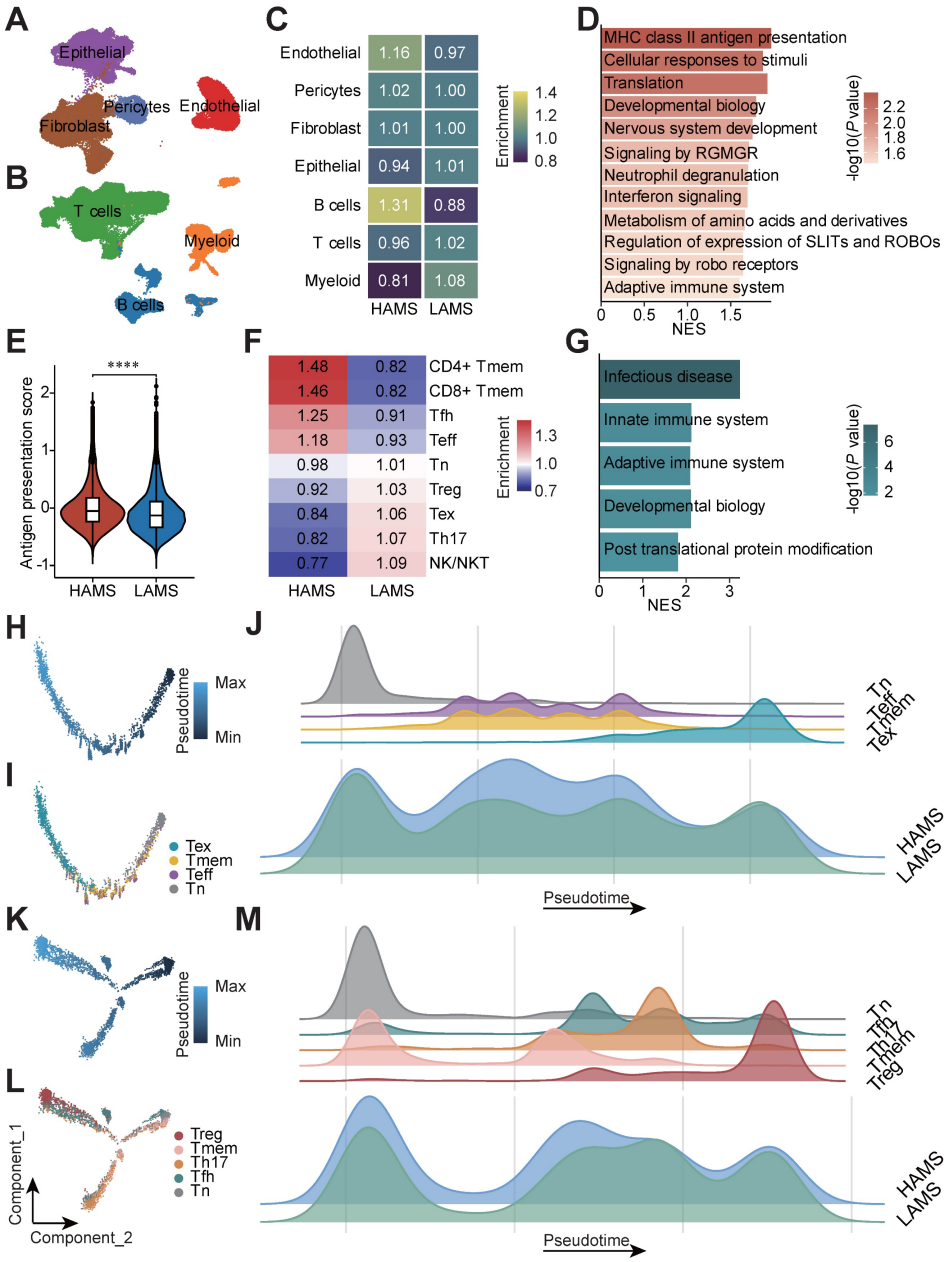
** Single-cell transcriptomic analysis deciphers that APOBEC mutagenesis activates immune response.** (**A-B**) UMAP plots of 44,340 CD45- (**A**) and 65,748 CD45+ (**B**) cells from 43 ESCC patients annotated by cell type. (**C**) Heatmap displaying the relative enrichment of each cell type in individuals with high and low AMS. (**D**) GSEA analysis of top 400 genes positively correlated with AMS in epithelial cells. (**E**) Violin plot showing antigen presentation score among different AMS groups. The *P* value of Wilcoxon rank sum test represented the significance. **** indicating *P* < 0.0001. (**F**) T Cells population enrichment differences between HAMS and LAMS group. (**G**) Results of GSEA in T cells showing functional pathway which genes positively correlated with AMS enriched in. (**H, K**) Pseudotime trajectory plots showing evolution of CD8+ T cells (**H**) and CD4+ T cells (**K**). Each dot representing a cell and the color intensity representing the pseudotime. (**I, L**) The developmental trajectory was plotted by subtype of CD8+ T cells (**I**) and CD4+ T cells (**L**). Each dot representing a cell and the color representing cell subtypes. (**J, M**) The density plots showing the distribution of CD8+ T cells (**J**) and CD4+ T cells (**M**) with different cell types along the pseudotime trajectory in patients with different AMS.

Moreover, we used our previously published scRNA-seq data from Cohort 1 to validate the conclusion 4. After quality control, a total of 110,088 cells including 44,340 CD45- and 65,748 CD45+ cells from 43 ESCC patients were analyzed and seven main cell types were identified: epithelial cells (18,791), fibroblast (17,004), endothelial cells (6,397), pericytes (2,148), T cells (43,892), B cells (11,847) and myeloid cells (10,009) (Figures 4A and 4B). Based on the optimal cut-off of AMS for OS of Cohort 1, these 43 ESCC patients were divided into HAMS and LAMS these two groups (Figures S3A and S3B). Great differences in the populations of these cell types were observed. Endothelia cells and B cells were enriched in the HAMS group, and the populations of myeloid cells were greater in the LAMS group (Figure 4C).

In consideration of epithelial cells being the core component of ESCC tumors, we focused on the transcriptome patterns of epithelial cells (Figure S3C). To elucidate the underlying biological diversities between the two groups, we found that genes positively correlated with AMS were significantly enriched in immune related pathways, such as MHC class II antigen presentation, IFN signaling and the adaptive immune system (Figure 4D). Then we calculated the antigen presentation score in epithelial cells and observed that the higher the AMS, the stronger the antigen presentation ability (Figure 4E and Table S3).

Since T cells were the most abundant cells in the tumor microenvironment in our data, we were particularly interested in T cell populations to evaluate the influence of APOBEC mutagenesis. We re-clustered T cells and observed nine subtypes of T cells: naïve T cells (Tn), T helper 17 cells (Th17), follicular helper T cells (Tfh), regulatory T cells (Treg), CD4+ T memery cells (CD4+ Tmem), CD8+ T memery cells (CD8+ T mem), effector T cells (Teff), exhausted T cells (Tex), and natural killer/natural killer T cells (NK/NKT) (Figures S3D and S3E). There were larger cell population in CD4+ Tmem, CD8+ Tmem, Tfh and Teff cells in HAMS group and Treg, Tex, Th17 and NK/NKT cells in the LAMS group (Figure 4F). The GSEA analysis showed that the innate and adaptive immune system pathway were probably significantly enriched in patients in the HAMS group (Figure 4G). According to analysis of signature genes, each T cell subset had distinct functional status. Patients in the HAMS group had lower exhaustion scores and regulation scores (Figures S3F, S3G and Table S3).

Furthermore, to visualize the developmental trajectory and imitate the cell evolution of CD4+ and CD8+ T cells, we performed pseudotime trajectory analysis in these two subsets of T cells (Figures 4H and 4K). The evolution trajectory originated from Tn cells and developed into Tmem and Teff cells, and finally turned into Tex cells for the CD8+ T cells and Tn cells developed into Tfh, Th17 and Tmem cells, and the Treg cells were the terminal state cells for the CD4+ T cells (Figures 4I and 4L). The density plot showed that the number of Teff and Tmem cells located in the middle stage of trajectory increased and Tex cells located in the late trajectory stage decreased in HAMS group (Figure 4J). Similarly, the number of Treg cells elevated in LAMS group (Figure 4M). All in all, our findings strongly elaborated that APOBEC mutagenesis could activate immunity by disrupting immune cell infiltration.

### The upregulation of A3A activity predominantly contributes to APOBEC mutagenesis in ESCC

**Figure 5:**
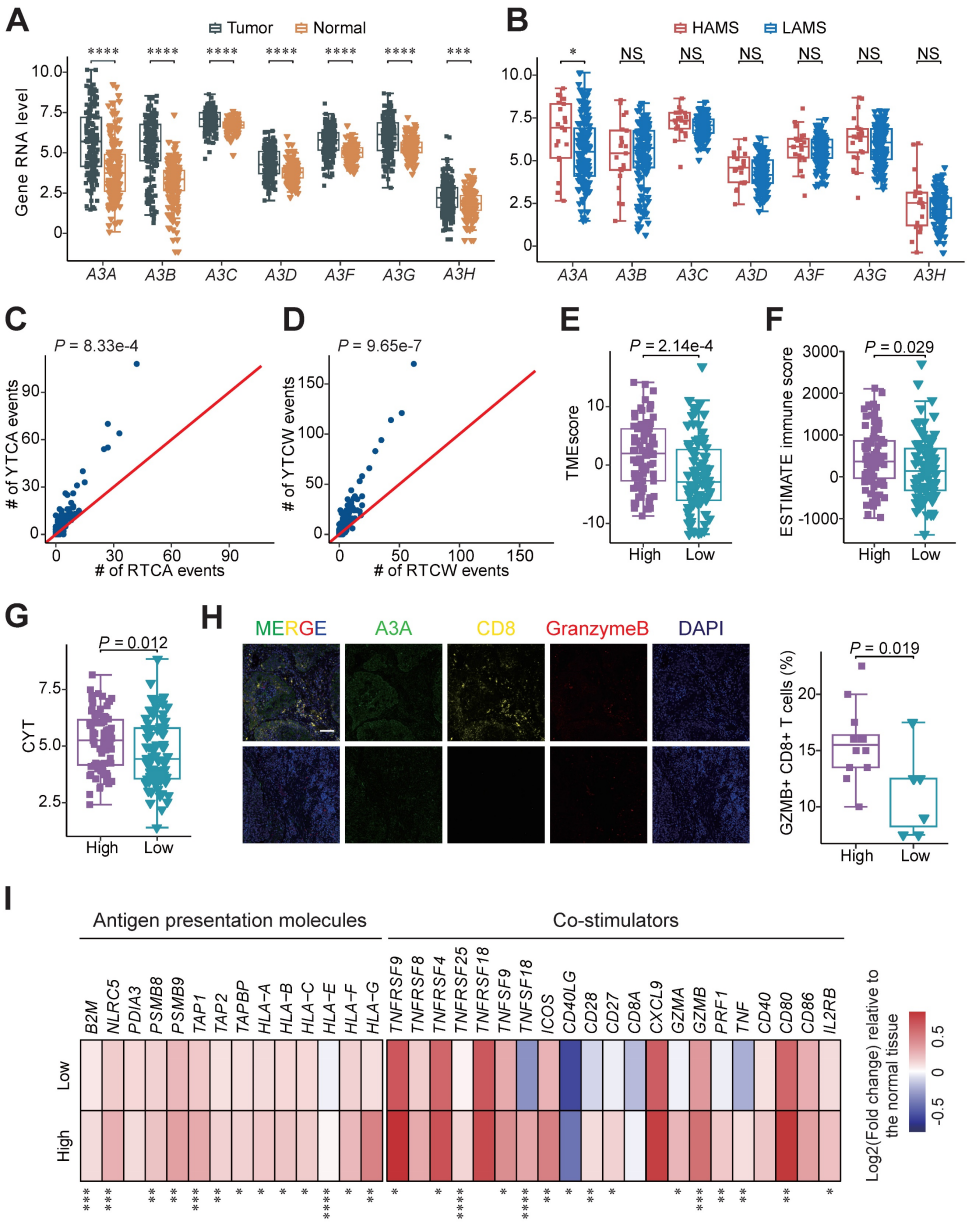
** A3A, the dominant mutator for APOBEC mutagenesis in ESCC, stimulates immune infiltration.** (**A-B**) Boxplots showing gene mRNA expression levels of APOBEC3 subfamily members between tumor and normal tissues (**A**) and tumors with high and low AMS (**B**). (**C-D**) Comparisons between counts of RTCA (or RTCW) events and YTCA (or YTCW) events. Red line indicating a 1:1 ratio. (**E-G**) Boxplots comparing the TMEscore (**E**), ESTIMATE immune score (**F**) and CYT score (**G**) between high and low *A3A* expression groups. Patients were divided into high and low groups based on the median *A3A* expression levels. (**H**) The left panel showing representative immunofluorescence images of Granzyme B+ CD8+ cells, representative of cytotoxic T cells, in tumors with different *A3A* expression levels. The boxplot in the right panel comparing the percentage of Granzyme B+ CD8+ T cells in tumors with different *A3A* expression levels from Cohort 1 (N = 19). Scale bar, 100 μm. (**I**) Heatmap displaying the log2 transformed fold change in tumors relative to that in normal tissues of antigen presentation molecules and immune stimulators. Boxplots displayed the median (central line), the 25-75% IQR (box limits), the ±1.5 times IQR (Tukey whiskers), respectively. The *P* value of Wilcoxon rank sum test represented the significance. * indicating *P <* 0.05, ** indicating *P <* 0.01, *** indicating *P <* 0.001, **** indicating *P <* 0.0001; and NS, not significant of two-sided Wilcoxon rank sum test.

The causal relationships between APOBEC3 subfamily members and mutational signatures in ESCC remain obscure, so we dissected which member is the major driver of APOBEC mutational signatures in ESCC. As shown in Figure 5A, all of the APOBEC3 members were dramatically elevated in tumor samples than adjacent normal tissues at RNA level. Those detected proteins, including A3A, A3B, A3F and A3G, were enriched in tumors at the protein level (Figures S4A and S4B) 46, 47. Then we compared the RNA expression levels of them in the two groups and found only *A3A* were increased in the HAMS group (Figure 5B). We further validated it using RT-qPCR approach in tumors from Cohort 1 (Figure S4C) 4. Among the 7 APOBEC3 members, *A3A* had the highest contribution to the performance of random forest model for AMS and TCW mutation counts, potentially suggested that APOBEC mutagenesis was associated with elevated *A3A* (Figures S4D and S4E). Because of the preference of mutation motifs for A3A and A3B 48, we compared the relative incidences of APOBEC-associated mutations in the RTCW motif relative to the YTCW motif in our cohort to further test the contribution of A3A to APOBEC mutagenesis in ESCC. We found that A3A-associated YTCA mutations were more abundant than A3B-associated RTCA mutations and the ratios of YTCA: RTCA and YTCW: RTCW were about 7:3, which is highly consistent with findings in oral squamous cell carcinoma (Figures 5C and 5D) 49. Altogether, the results from our integrated analyses revealed that A3A was the major mutator contributing to the APOBEC mutagenesis in ESCC.

### *A3A* promotes immune response by activating cGAS-STING pathway

After demonstrating A3A is the dominant contributor to the APOBEC mutagenesis, we performed survival analyses and compared the immune-related score between patients with different expression levels of *A3A* to verify the clinical relevance and functional impact of *A3A*. As expected, Kaplan-Meier survival analysis revealed that OS rates were higher in patients with high *A3A* RNA levels than these with low *A3A* RNA levels (Figures S4F-I). Compared to low *A3A* group, the TMEscore and ESTIMATE immune score were increased in the high *A3A* group (Figures 5E and 5F). We also consistently observed the expression of immune regulation related molecule *IFNG* was enhanced as the *A3A* expression increased, as well as the CYT score (Figures S4J and 5G). Multiplex immunofluorescence analysis verified that *A3A* overexpression greatly facilitated the infiltration of cytotoxic T cells in ESCC tissues (Figure 5H). The expression of immunomodulatory molecules such as antigen presentation molecules and co-stimulators were employed to compare between the high and low *A3A* groups. Majority of these molecules were significantly increased in the high *A3A* group (Figure 5I). These results elaborated that *A3A* is the pivotal molecule for bridging high AMS with abundant immune filtration and better survival.

**Figure 6:**
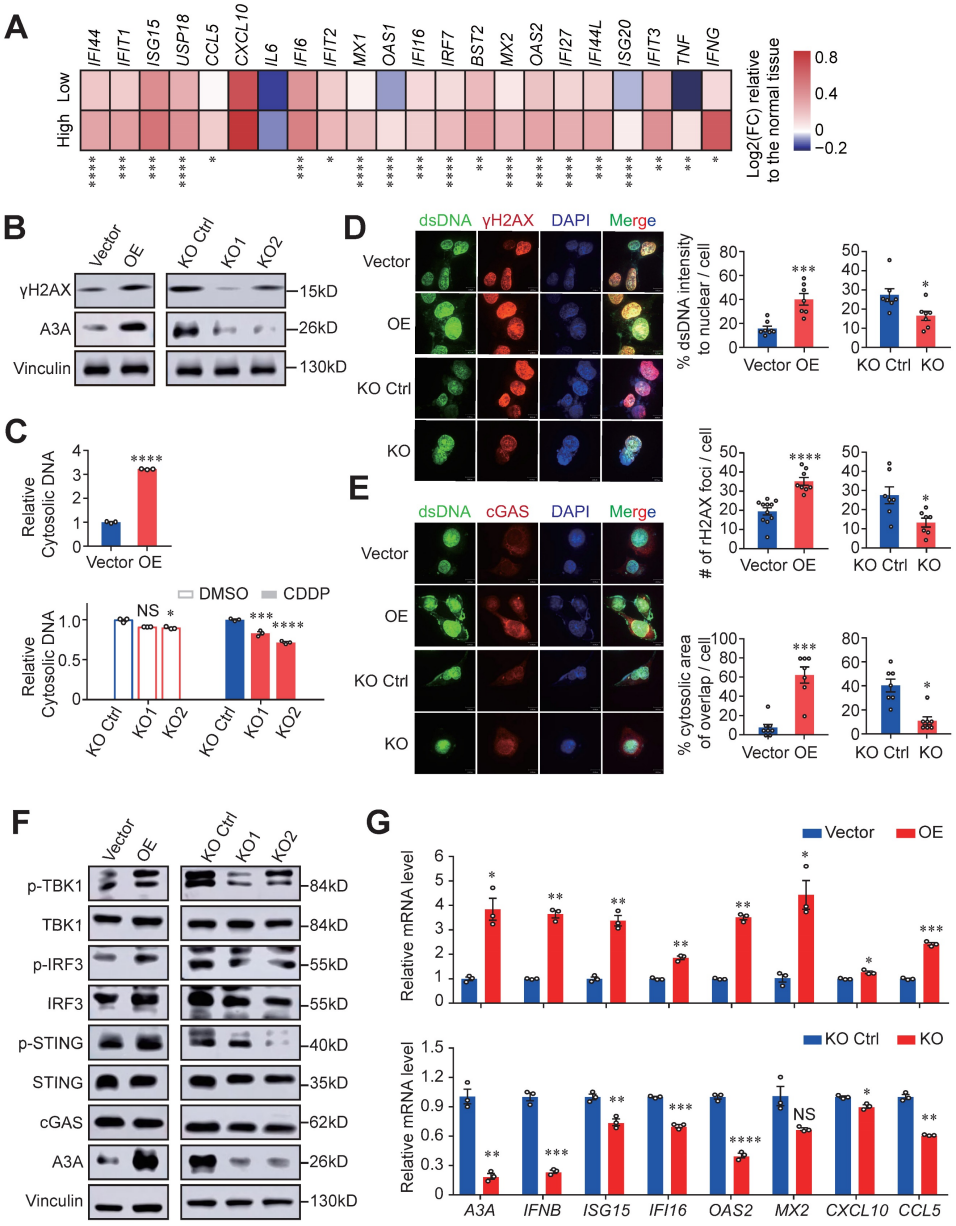
***A3A* overexpression enhances immune signaling by activating cGAS-STING pathway.** (**A**) Heatmap showing the differential expressions of ISGs between the high and low *A3A* groups. FC: fold change. (**B**) Western blot analysis of A3A and γH2AX levels of KYSE30 with *A3A* overexpression (OE) or knockout (KO). (**C**) Cytosolic dsDNA isolated by a commercial kit and quantified in KYSE30 with *A3A* OE. Cytosolic dsDNA also quantified in KYSE30 with *A3A* KO after treated with CDDP or DMSO. (**D-E**) Representative confocal microscopy images (left) of dsDNA, γH2AX and cGAS in the KYSE30 with *A3A* OE or KO. Statistical graphs (right) showing the proportion of extra-nuclear dsDNA, quantitative analysis of γH2AX foci and the area of cytoplasmic cGAS overlapped with cytosolic dsDNA. KYSE30 with *A3A* KO were treated with CDDP to induce DNA damage. Scale bars, 10 μm. (**F**) Western blot analysis of key factors in cGAS-STING pathway including total and p-TBK1, total and p-IRF3, total and p-STING and cGAS in KYSE30 with *A3A* OE or KO. (**G**) RT-qPCR quantifying *A3A*, *IFNB* and several representative ISGs levels, including *ISG15*,* IFI16*,* OAS2*,* MX2*, *CXCL10* and *CCL5*, in KYSE30 with *A3A* OE or KO. Data are shown as mean ± SEM. * indicating *P <* 0.05, ** indicating *P <* 0.01, *** indicating *P <* 0.001, **** indicating *P <* 0.0001, and NS, not significant of Student's t-test.

Next, we investigated the underlying mechanisms of immune activation accompanied by high *A3A* levels. Based on the above suggestive results that type I and II IFN signaling, essential drivers of anti-tumor immunity, were upregulated and might have a vital role in APOBEC mutagenesis mediated immune activation, we measured the expression of IFN-stimulated genes (ISGs) in different *A3A* level groups and found that most of ISGs were overexpressed in patients with high *A3A* levels (Figure 6A). As the stimulator of IFN genes, the cGAS-STING pathway has emerged as a key regulator of innate and adaptive immune and can be activated by cytoplasmic dsDNA, we therefore measured the abundance of DNA damage-induced DSBs and cytosolic dsDNA. We found that both γH2AX and cytosolic dsDNA levels were significantly elevated in cells overexpressing *A3A* while declined in KYSE30 and KYSE510 cells with *A3A* silenced (Figures 6B, 6C, S5A and S5B), which were verified by immunofluorescence staining (Figures 6D and S5C). Due to the relatively low basal levels of DSBs and DSBs-induced cytosolic dsDNA, no obvious decline appears in *A3A* knockout cells. Hence, we treated KYSE30 and KYSE510 cells with a commonly used chemotherapy agent CDDP to boost DNA damage.

Indeed, cytosolic dsDNA and γH2AX levels significantly decreased in *A3A* knockout cells treated with CDDP (Figures 6C, 6D, S5B and S5C). To determine whether the accumulated cytosolic dsDNA activate immunity mediated by cGAS-STING pathway, we assessed the presence of cytosolic dsDNA and cGAS, which displayed co-localization in cytoplasm (Figures 6E and S5D). *A3A* overexpression remarkably increased the interplay between dsDNA and cGAS in KYSE30 and KYSE510 cells. In contrast, abolishing of *A3A* decreased the co-localization of these two molecules (Figures 6E and S5D). These data indicated that dysregulation of *A3A* could potentially stimulate cGAS pathway and its downstream factors. Consistently, *A3A* overexpression cells contained higher levels of p-STING, p-IRF3 and p-TBK1 (Figures 6F and S5E), as well as the downstream ISGs upregulation (Figures 6G and S5F-S5H). However, silencing of *A3A* impaired the pathway signaling (Figures 6F, 6G, and S5E-S5H). Altogether, we concluded that *A3A* increased the accumulation of cytosolic dsDNA by causing robust DNA damages, which subsequently activate cGAS-STING pathway and then potentiate anti-tumor immune signaling.

### Dysregulation of *A3A* is resulted from the overexpression of transcription factor FOSL1

**Figure 7:**
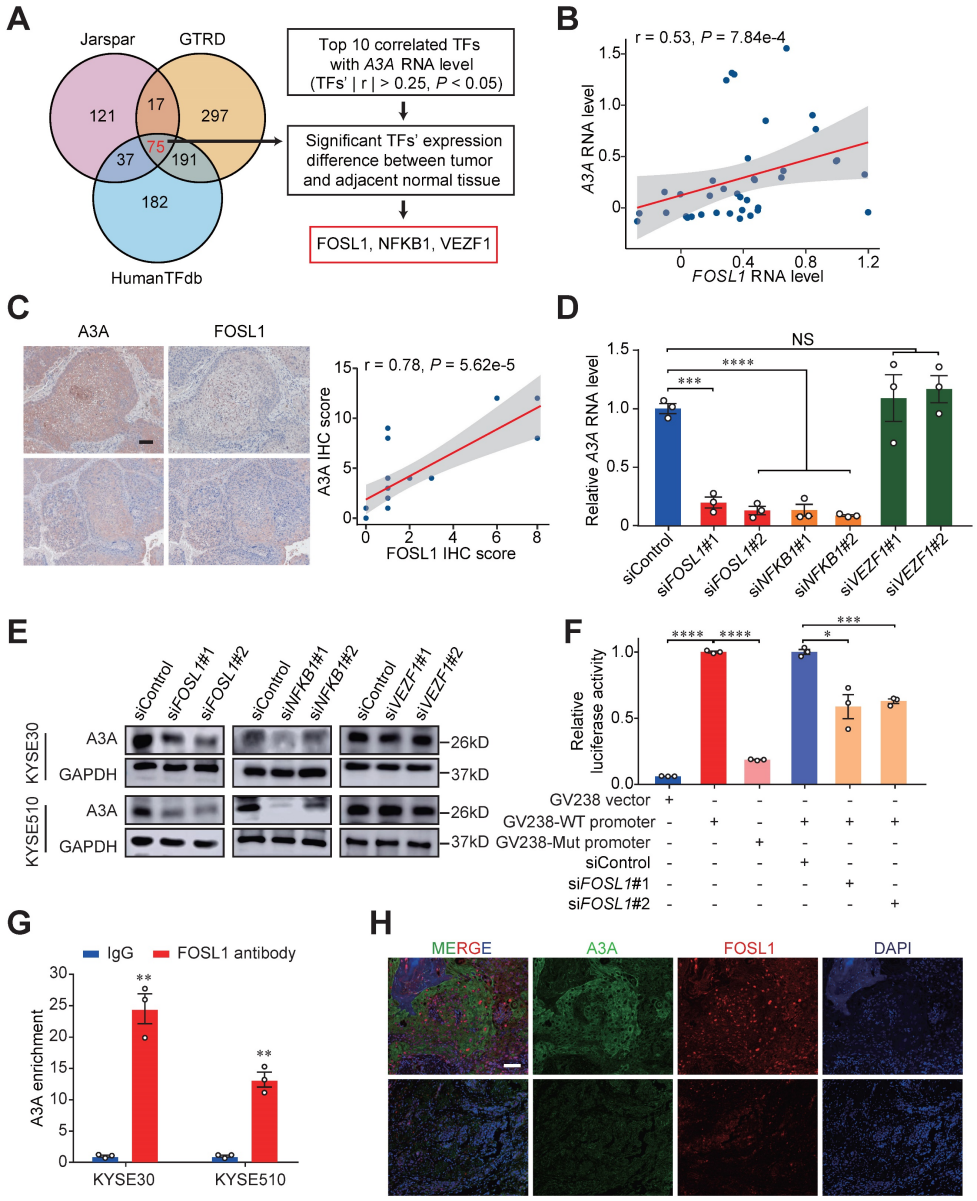
** FOSL1, a key transcription factor, promotes the expression of *A3A*.** (**A**) Venn diagram and flow chart showing the prediction process of the three TFs. (**B**) Spearman correlation between *FOSL1* and *A3A* RNA levels in scRNA-seq data. (**C**) Representative IHC staining photomicrographs displaying the correlation between FOSL1 and A3A protein levels (N = 20, tissues collected from Cohort 1). Scale bar, 100 μm. (**D**) RT-qPCR showing the influence of the indicated TFs knockdown on *A3A* RNA level in KYSE30. (**E**) Western blot analysis demonstrating the influence of the indicated TFs knockdown on A3A protein levels in KYSE30 and KYSE510. (**F**) Dual luciferase reporter assays showing elevated luciferase activity in KYSE510 transfected with GV238-WT promoter and decreased activity after knockdown *FOSL1* by siRNA. (**G**) ChIP-qPCR determination showing *A3A* mRNA enrichment in cell lysates treated with FOSL1 antibody in KYSE30 and KYSE510. (**H**) Representative multiplexed immunofluorescent staining images showing the positive correlation and co-localization between A3A and FOSL1. Scale bar, 100 μm. Data are shown as mean ± SEM. * indicating *P <* 0.05, ** indicating *P <* 0.01, *** indicating *P <* 0.001, **** indicating *P <* 0.0001, and NS, not significant of Student's t-test.

After ensuring that APOBEC mutagenesis was caused by elevated *A3A* expression levels in ESCC, we sought to investigate the potential mechanism of dysregulation of *A3A* expression. We first examined the associations between *A3A* expression levels and copy number variations in Cohort 2 and TCGA-Asian cohort but found no significant correlation (Figure S6A). Then the methylation levels of *A3A* was taken into consideration and no significant conclusion was drawn (Figure S6B). Next, we performed in silico analyses to interrogate potential TF binding sites using the promoter sequence of *A3A* in three public databases, including Jarspar, GTRD and HumanTFdb, and 75 TFs overlapped by the three databases were obtained (Figure 7A). Among the top 10 significantly correlated TFs with *A3A* RNA levels in bulk RNA-seq profiles, three TFs' (*FOSL1*, *NFKB1* and *VEZF1*) expression levels were greatly different between tumor and normal tissues. We therefore speculated that FOSL1, NFKB1 and VEZF1 were the most credible TFs resulting in the overexpression of *A3A*. Finally, we validated the co-expression relationship of the three TFs and *A3A* in scRNA-seq data and found only *FOSL1* was significantly co-expressed with *A3A* (Figures 7B, S6C and S6D). IHC staining further verified the result (Figure 7C).

To find out which TF regulating the expression of *A3A*, we examined the RNA and protein levels of *A3A* in cells with knockdown of these three TFs, and found both siRNA targeting *FOSL1* and *NFKB1* suppressed the *A3A* expression (Figures 7D, 7E and S6E-H)*.* The nuclear factor of kappa light chain enhancer of activated B cells (NF-κB) is a well-known transcription factor, which represents a family of structural and functional related proteins (NFκB1, NFκB2, RelA, RelB and c-Rel). Among them, NFκB1, binding with gene promoters, exists as homo- or hetero- dimer with all NF-κB transcription factor family members 50. NF-κB (RelA) repsonse was found to regulate the expression of *A3A* 51. To find a novel way to explain the dysregulation of *A3A*, we therefore hypothesized that FOSL1 was another causal TF of *A3A*. To elucidate the effects of FOSL1 on the *A3A* expression, we carried out luciferase reporter assays and ChIP-qPCR assays. The potential TF binding site was shown in Figure S6I. The results indicated that the plasmid construct containing wild type promoter sequence had increased luciferase activity compared with mutant and empty counterparts. What's more, luciferase activity significantly decreased when knocking down *FOSL1* in cells transfected with plasmid construct containing wild type promoter sequence (Figure 7F). ChIP-qPCR assays showed that FOSL1 could bind to *A3A* promoter (Figure 7G). We also conducted immunofluorescence staining of FOSL1 and A3A and found the co-localization and positive correlated expression pattern of these two molecules (Figure 7H). Together, FOSL1 was supposed to be a transactivator regulating *A3A* expression in ESCC.

### *A3A* overexpression upregulates STAT1 signaling and predicts immunotherapy response

**Figure 8:**
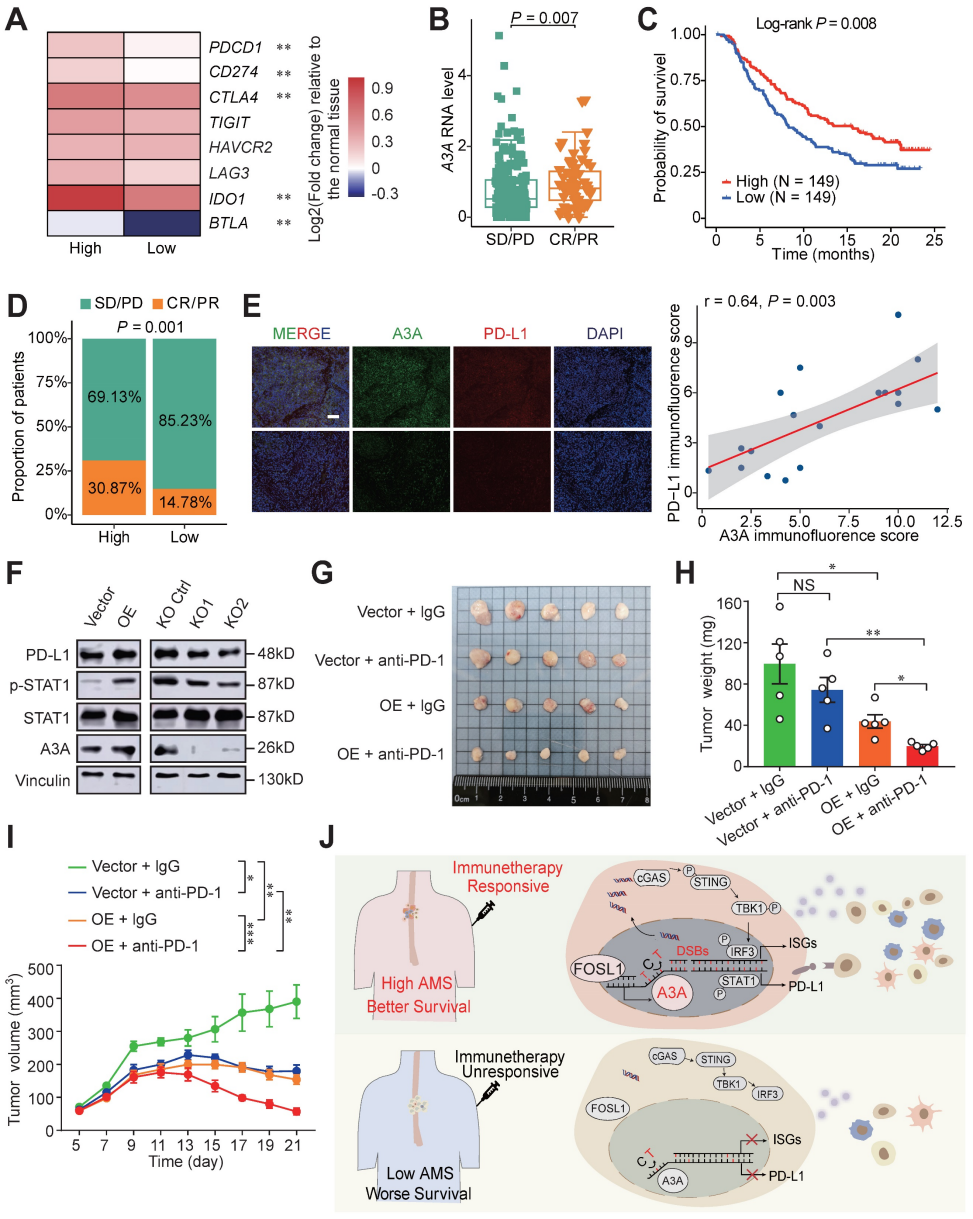
***A3A*'s role in predicting immunotherapy response.** (**A**) Heatmap showing the log2 transformed fold change in tumors relative to that in normal tissues of several important immune checkpoints. The *P* value of Wilcoxon rank sum test represented the significance. ** indicating *P <* 0.01 of two-sided Wilcoxon rank sum test. (**B**) *A3A* mRNA levels comparison between patients with different anti-PD-L1 treatment response. (**C**) The Kaplan-Meier survival curves according to *A3A* mRNA levels in patients receiving anti-PD-L1 treatment. (**D**) The proportion of patients with different treatment response in high and low *A3A* groups. (**E**) Representative multiplexed immunofluorescent staining pictures showing the positive correlation and co-localization between A3A and PD-L1 (N = 19, tissues collected from Cohort 1). Scale bar, 100 μm. (**F**) Western blot analysis of PD-L1, total and p-STAT1 in KYSE30 with *A3A* OE or KO. (**G**) Image of the mouse tumors with or without *A3A* overexpression receiving anti-PD-1 treatment or IgG control treatment at the end of the experiment. (**H-I**) Statistical graph showing the weight of subcutaneous tumors (**H**) and tumor growth curves showing the tumor volume (**I**) among the four groups (N=5 per group). Data are shown as mean ± SEM. * indicating *P <* 0.05, ** indicating *P <* 0.01, *** indicating *P <* 0.001, **** indicating *P <* 0.0001, and NS, not significant of Student's t-test. (**J**) A proposed model for the regulatory mechanism of APOBEC mutagenesis in immunity and immunotherapy response in ESCC.

Since tumor-infiltrating lymphocytes density in tumors were demonstrated critically correlated with the immunotherapy response, we therefore examined the predictive potential and the mechanism of *A3A* for ESCC immunotherapy 20, 52. The expression of eight immune checkpoint molecules was compared between high and low *A3A* groups. Five molecules (*PDCD1*, *CD274*,* CTLA4*, *IDO1* and *BTLA*) were significantly upregulated in high *A3A* group (Figure 8A). Furthermore, the low *A3A* group presented higher TIDE score, indicating worse immunotherapy response and the fraction of patients with CR/PR (complete response/partial response) in high *A3A* group was significantly increased than low *A3A* group (Figures S7A, B). More critically, we ultimately employed a urothelial carcinoma dataset, called IMvigor210, receiving anti-PD-L1 therapy to evaluate *A3A* predictive ability for immunotherapy 53.

Patients with CR or PR response displayed elevated *A3A* expression levels than patients with SD (stable disease) or PD (progression disease) response (Figure 8B). The high *A3A* patients possessed prolonged survival time and higher percentage of patient with better response than low *A3A* group (Figures 8C, D). PD-L1 expression enhanced by *A3A* overexpression was confirmed by immunofluorescence and Western blot analysis (Figures 8E and 8F). Previous reports have shown that DSBs could induce STAT1 signaling leading to PD-L1 upregulation 54, we thus explored whether *A3A* overexpression could stimulate STAT1 signaling to promote the expression of PD-L1. We found that p-STAT1 levels, not total STAT1, were increased in cells overexpressing *A3A* but declined in cells with *A3A* knockout (Figure 8F). Because we found that *A3A* prompted anti-tumor infiltration and important immune checkpoints expression, we next examined whether *A3A* was involved in sensitizing to immunotherapy in vivo in ESCC. C57BL/6 mice were subcutaneously injected mEC25 cells with *A3A* overexpression or not and treated with anti-PD-1 therapy or IgG isotype control antibody (Figure S7C). We found that *A3A* overexpression blunted tumor growth in C57BL/6 mice (Figure S7D-F). Unsurprisingly, the tumor volume and weight significantly decreased in *A3A* overexpression group compared with control group after anti-PD-1 treatment, which indicating that *A3A* overexpression enhanced immunotherapy efficacy (Figure 8G-I). Overall, these data raised the prospect of *A3A* in predicting immunotherapy response.

## Discussion

APOBEC mutational signatures are detected in at least 22 different cancer types and reported to be near-universally exhibited in ESCC 5, 24, but the functional effects of APOBEC mutagenesis so far are ambiguous in ESCC. In the present study, we conduct integrated analyses based on genomic profiles, bulk and single cell transcriptomic profiles of 169 patients with ESCC and a set of functional experiments to address this issue. These analyses reveal that APOBEC mutagenesis remarkably potentiated the anti-tumor effects by augmenting immune cell infiltration. Then we have focused on which one of APOBEC3 subfamily member is linked with the APOBEC related mutation pattern. On the basis of the mutational sequence and APOBEC3s' expression pattern in ESCC, we conclude that APOBEC mutagenesis may be contributed by *A3A* upregulation. Furthermore, the dysregulation of *A3A* is due to FOSL1. As expected, *A3A* overexpression aggravate DNA damage and DSBs, which promotes leaking of nuclear DNA into the cytoplasm, leading to accumulation of cytosolic dsDNA. Cytosolic dsDNA sensed by cGAS stimulate cGAS-STING signaling to trigger anti-tumor immune response, which results in better survival. In addition, *A3A* improves PD-L1 expression mediated by STAT1 signaling. Two aspects mentioned above make patients enriched with APOBEC mutational signatures benefit from immunotherapy (Figure 8I).

APOBEC3 gene cluster located at 22q13 in human genome, which encode members of a superfamily of cytidine deaminases that convert cytidines to uracils (C-to-U) in ssDNA. Its enzymatic activity has been implicated in diverse biological functions, including innate and adaptive immune responses and viral restriction 55, 56. Mutational patterns of A3A and A3B are apparently distinguishable. A3A favors YTCA sites and A3B favors RTCA sites 48. Several studies have reported that A3B is a putative mutation driver to initiate the main source of somatic mutations and associated with poor survival 57, 58. Ting-Wen and colleagues claimed overpresentation of APOBEC mutational signatures resulted from altered tumor-related *A3A* expression in Taiwanese oral squamous cell carcinoma 49. Consistent with the previous findings, emerging evidences have identified A3A as the main mutator to these mutations in bladder carcinoma, breast cancer, or even in recessive dystrophic epidermolysis bullosa 7, 59. Specifically, the current study reveals a causal links between *A3A* and APOBEC mutagenesis and demonstrate improved OS in ESCC patients with high levels of *A3A*, which might due to the increased immune infiltration. The multifaceted observations may be explained by heterogeneity among different cancer types.

APOBEC mutagenesis inhibits breast cancer growth through immune activation and correlate with immune infiltration in bladder cancer 60, 61. On the contrary, it is reported that APOBEC mutagenesis fuels the subclones evolution resulting in drug resistance and immune escape 62, 63. The controversial results hint that the direct functions of APOBEC mutagenesis in tumor microenvironment are not entirely known. To our best known, our study is the first to explore the mechanism of APOBEC mutagenesis mediating immune activation in ESCC. We have delineated that APOBEC mutagenesis promotes activation of cGAS-STING pathway and upregulation of IFN and the downstream ISGs such as *CXCL10* and *CCL5*. CXCL10 and CCL5 are vital chemokines for CD8+ T lymphocytes, the upregulation of which increase the abundance of CD8+ T lymphocytes 64. *CXCL10* and *CCL5* mRNA levels increase in patients with high *A3A* levels compared with low *A3A* group, although *CXCL10* does not achieve the significance level in our bulk RNA-seq data (Figure 6A). The RT-qPCR results showed significant difference for the two molecules (Figure 6G and S5F). Therefore, cancer cells overexpressing *A3A* augment anti-tumor immune response might by secreting CCL5 and CXCL10 to recruit and activate CD8+ T cells in ESCC, which warrants further functional investigations.

Studies have recapitulated that anti-tumor response is more obvious in PD-L1 positive patients with advanced and metastatic esophageal cancer treated with anti-PD-1 agents 65, 66, as well as non-small cell lung carcinoma (NSCLC) treated with anti-PD-L1 therapy 67, 68. Pre-existing CD8+ T lymphocytes is also an indicator of effective immunotherapy 20. Immunotherapy has occupied an essential position for clinical therapeutic strategies of ESCC. Our research raises the prospect of *A3A* utility for immunotherapy response prediction for ESCC using bioinformatic analyses and validate it in mouse models and clinical data. Furthermore, we found elevated immune infiltration and PD-L1 levels in *A3A* overexpression cells and explained the possible mechanisms using functional experiments.

Interestingly, we have identified a new TF, FOSL1, transactivating expression of *A3A*. FOSL1 has been identified as core regulatory circuitry candidates for ESCC 69. Although FOSL1 is highly expressed in most tumors and promotes malignant progression in malignant tumor 70, 71, but it may be at the core of T cell differentiation 72. FOSL1 is actively involved in cytokines secretion, such as IL-6, which inhibits Treg differentiation and induces CD8+ T cells to differentiate into cytotoxic T cells 73. These results emphasize that FOSL1 may be the key upstream factor of *A3A* and plays a crucial role in APOBEC mutagenesis induced immunity in ESCC.

In short, patients enriched with APOBEC mutagenesis are characterized with longer OS, higher anti-tumor and lower immune-inhibitory immune infiltration and elevated immune checkpoint expression, which suggests that APOBEC mutagenesis is greatly associated with prognosis and response to ICB treatment. What's more, scRNA-seq data analyses and functional assays were performed to elucidate the potential mechanisms. However, APOBEC mutagenesis plays a double-edged sword role in cancer. A critical cutoff value and the role of APOBEC mutagenesis in assessing prognosis and the immunotherapy responses of ESCC remain to be accurately determined through prospective studies.

## Conclusions

In conclusion, we illustrated whether and how APOBEC mutagenesis activates immune response using WGS/WES, bulk RNA-seq, scRNA-seq data and functional assays. These findings uncovered a new biomarker to accelerate the clinical application and improve immunotherapy effects in ESCC.

## Supplementary Material

Supplementary figures and tables.

## Abbreviations

APOBEC: apolipoprotein B mRNA editing enzyme catalytic polypeptide
ESCC: esophageal squamous cell carcinoma
scRNA-seq: single-cell RNA sequencing
OS: overall survival
IFN: interferon
A3A: APOBEC3A
dsDNA: double-stranded DNA
A3B: APOBEC3B
A3C: APOBEC3C
A3D: APOBEC3D
A3F: APOBEC3F
A3G: APOBEC3G
A3H: APOBEC3H
ssDNA: single-stranded DNA
RNA-seq: RNA sequencing
TF: transcription factor
FOSL1: FOS Like 1
COSMIC: Catalogue of Somatic Mutations in Cancer
AMS: APOBEC mutagenesis enrichment score
FDR: false discovery rate
BH: Benjamin-Hochberg
HAMS: high AMS
LAMS: low AMS
GSEA: gene set enrichment analysis
GSVA: gene set variation analysis
ICB: immune checkpoint blockade
GEO: Gene Expression Omnibus
PCA: principal component analysis
CDDP: cis-dichlorodiammineplatinum
DMSO: dimethylsulfoxide
p-STING: phospho-STING
p-IRF3: phospho-IRF3
p-TBK1: phospho-TBK1
p-STAT1: phospho-STAT1
IHC: immunohistochemistry
FFPE: formalin-fixed paraffin-embedded
siRNA: small interfering RNA
WT: wild-type
Mut: mutant
ChIP: chromatin immunoprecipitation
ChIP-qPCR: ChIP-quantitative real-time PCR
SNVs: single nucleotide variations
TMB: tumor mutation burden
DDR: DNA damage repair
HRs: hazard ratios
CI: confidence interval
WGS: whole genome sequencing
WES: whole exome sequencing
DSBs: double-stranded breaks
Tn: naïve T cells
Th17: T helper 17 cells
Tfh: follicular helper T cells
Treg: regulatory T cells
Tmem: T memery cells
Teff: effector T cells
Tex: exhausted T cells
NK/NKT: natural killer/natural killer T cells
ISGs: IFN-stimulated genes
NF-κB: nuclear factor of kappa light chain enhancer of activated B cells
CR: complete response
PR: partial response
SD: stable disease
PD: progression disease
C-to-U: cytidines to uracils
NSCLC: non-small cell lung carcinoma
