# Supplementary material for: Comprehensive Analyses Reveal Effects on Tumor Immune Infiltration and Immunotherapy Response of APOBEC Mutagenesis and Its Molecular Mechanisms in Esophageal Squamous Cell Carcinoma
Source: Int J Biol Sci. 2023 May 8;19(8):2551–71. doi: 10.7150/ijbs.83824 (PMC10197887; doi:10.7150/ijbs.83824)
Supplement: Supplementary file 1 — Supplementary figures and tables. [file ijbsv19p2551s1.pdf]

Figure S1. Mutational spectrum of ESCC, related to Figure 1. (**A**) Relative abundance of six types of single base substitutions (C>A, C>G, C>T, T>A, T>C, T>G) in ESCC. The x-axis denoting the 16 possible trinucleotide contexts repeated for each category. (**B**) Proportion differences of mutations occurring in TCW motifs between HAMS and LAMS group. The specific cutoff to dichotomize AMS was based on the minimum log-rank *P* in survival analysis. (**C**) The oncoplots of genes in DDR pathways. The top left panel showing the landscape of nonsynonymous mutations in DDR genes. The top right bar plot indicating the number of mutated patients for each gene. And the bottom panel displaying six categories of single base substitutions in DDR genes.

Figure S2. Transcriptomic features associated with APOBEC mutagenesis, related to Figure 3. (**A-C**) Correlations between AMS and the expression levels of representative genes in three pathways (IFN signaling, innate immune system and MHC class II antigen presentation). (**D**) Specific pathway activity comparison measured by GSVA scores between patients with upper and lower quantile of AMS. (**E-H**) Boxplots displaying StromalScore, TumorPurity, several immune checkpoints expression levels and CYT score among upper and lower quantile of AMS groups. Boxplots displayed the median (central line), the 25–75% IQR (box limits), the ±1.5 times IQR (Tukey whiskers), respectively. The *P* value of Wilcoxon rank sum test represented the significance. * indicating $P < 0.05$, ** indicating $P < 0.01$.

Figure S3. Cell distribution and expression profiles of epithelial and T cells. Data related to Figure 4. (**A**) UMAP plot of CD45+ cells (left) and CD45- cells (right) in patients with different level of AMS. Color represented cell types and was identical with in Figure 4A and 4B; (**B**) Dotplots showing the average expression levels of marker genes (color intensity) and fraction of cells expressed (circle size) in CD45- (top) and CD45+ (bottom) cells. (**C**) UMAP plot of epithelial cells, colored by patients. (**D**) UMAP plot of all T cells, colored by T

cell subtypes. (**E**) Heatmap showing the average expression levels of marker genes of each subtype of T cells. (**F-G**) Violin plots comparing the regulation score and exhaustion score between tumors with HAMS and LAMS. The *P* value of Wilcoxon rank sum test represented the significance. **** indicating $P < 0.0001$.

Figure S4. A3A is the most important contributor to APOBEC mutagenesis and confers a protective effect in ESCC. Data related to Figure 5. (**A-B**) Comparison of protein levels of APOBEC3 enzymes between tumor and normal tissues (**A**) or paired tumor and normal tissues (**B**). Data was collected from two published databases. (**C**) The results of comparing mRNA levels of APOBEC3 genes between HAMS and LAMS determined by RT-qPCR. (**D-E**) IncnodePurity of random forest was used to identify the relative importance of APOBEC3 genes for AMS (**D**) and TCW mutations (**E**). (**F-I**) The Kaplan-Meier survival curves according to *A3A* expression in three independent cohorts (Cohort 1, Cohort 2 and TCGA-Asian), and combined cohort 2 combing the three cohorts. *P* values were derived from log-rank test. HR and 95% CI derived from multivariate Cox proportional hazard models adjusting age, gender, clinical stage, smoking and drinking status were presented. (**J**) Boxplot showing the *IFNG* mRNA levels among patients with different *A3A* levels. Boxplots displayed the median (central line), the 25–75% IQR (box limits), the ±1.5 times IQR (Tukey whiskers), respectively. The *P* value of Wilcoxon rank sum test represented the significance. * indicating $P < 0.05$, ** indicating $P < 0.01$, *** indicating $P < 0.001$, **** indicating $P < 0.0001$, and NS, not significant of two-sided Wilcoxon rank sum test.

Figure S5. *A3A* stimulates immune response mediated by cGAS-STING pathway, related to Figure 6. (**A**) Western blot analysis of A3A and γH2AX levels of KYSE510 with *A3A* OE or KO. (**B**) Cytosolic dsDNA isolated by a commercial kit and quantified in KYSE510 with *A3A* OE. Cytosolic dsDNA also

quantified in KYSE510 with *A3A* KO after treated with CDDP or DMSO. (**C-D**) Representative confocal microscopy images (left) of dsDNA, γH2AX and cGAS in the KYSE510 with *A3A* OE or KO. Statistical graphs (right) showing the proportion of extranuclear dsDNA, quantitative analyses of γH2AX foci and the area of cytoplasmic cGAS overlapped with cytosolic dsDNA. KYSE510 with *A3A* KO were treated with CDDP to induce DNA damage. Scale bar, 10 μm. (**E**) Western blot analysis of key factors in cGAS-STING pathway including total and p-TBK1, total and p-IRF3, total and p-STING and cGAS in KYSE510 with *A3A* OE or KO. (**F**) RT-qPCR quantifying *A3A*, *IFNB* and several representative ISGs levels, including *ISG15*, *IFI16*, *OAS2*, *MX2*, *CXCL10* and *CCL5* in KYSE510 with *A3A* OE or KO. (**G-H**) RT-qPCR quantifying other ISGs levels, including *IFNG*, *IFIT2*, *IFIT3*, *IFI6*, *IFI27* and *OAS1*, in KYSE30 (**G**) and KYSE510 (**H**) with *A3A* OE or KO.

Data are shown as mean ± SEM. * indicating $P < 0.05$, ** indicating $P < 0.01$, *** indicating $P < 0.001$, **** indicating $P < 0.0001$, and NS, not significant of Student's t-test.

Figure S6. Identification of FOSL1 as the transcription factor of *A3A*. Data related to Figure 7. (**A**) Spearman correlations between *A3A* copy number and its RNA level in Cohort 2 (left) and TCGA-Asian cohort (right). (**B**) Spearman correlations between the *A3A* methylation levels and its RNA level in Cohort 2 (left) and TCGA-Asian cohort (right). (**C-D**) Spearman correlations between *NFKB1* (**C**) and *VEZF1* (**D**) with *A3A* RNA levels in scRNA-seq data. (**E-G**) RT-qPCR verifying the knockdown of indicated TFs by siRNA in KYSE30 and KYSE510 cell lines. (**H**) RT-qPCR showing the influence of the indicated TF knockdown on *A3A* RNA levels in KYSE510. (**I**) The sequences of *A3A* wildtype promoter and the FOSL1-binding motif-deletion mutant for the GV238 reporter gene plasmid constructions.

Data are shown as mean ± SEM. * indicating $P < 0.05$, ** indicating $P < 0.01$, *** indicating $P < 0.001$, **** indicating $P < 0.0001$ and NS, not significant of

Student's t-test.

Figure S7. *A3A* engaging in slowing tumor growth and enhancing immunotherapy efficacy. Data related to Figure 8. (**A**) Comparison of TIDE score between high and low *A3A* groups. Boxplots displaying the median (central line), the 25–75% IQR (box limits), the ±1.5 times IQR (Tukey whiskers), respectively. (**B**) The bar graph illustrating the prediction of treatment response in high and low *A3A* groups. (**C**) Schematic illustration of the mouse model construction. Mice were subcutaneously injected with mouse ESCC cells, named mEC25, with or without *A3A* overexpression (upper). Anti-PD-1 and the IgG control antibody were intra-tumoral injected at the indicated time points (lower). (**D**) Image of the mouse tumors with or without *A3A* overexpression at the end of the experiment. (**E-F**) Statistical graph showing the tumor growth curves showing the tumor volume (**E**) and weight of subcutaneous tumors (**F**) between the two groups (N=5 per group). Data are shown as mean ± SEM. * indicating $P < 0.05$, ** indicating $P < 0.01$ of Student's t-test.

Yang et al. Supplementary Figure 1

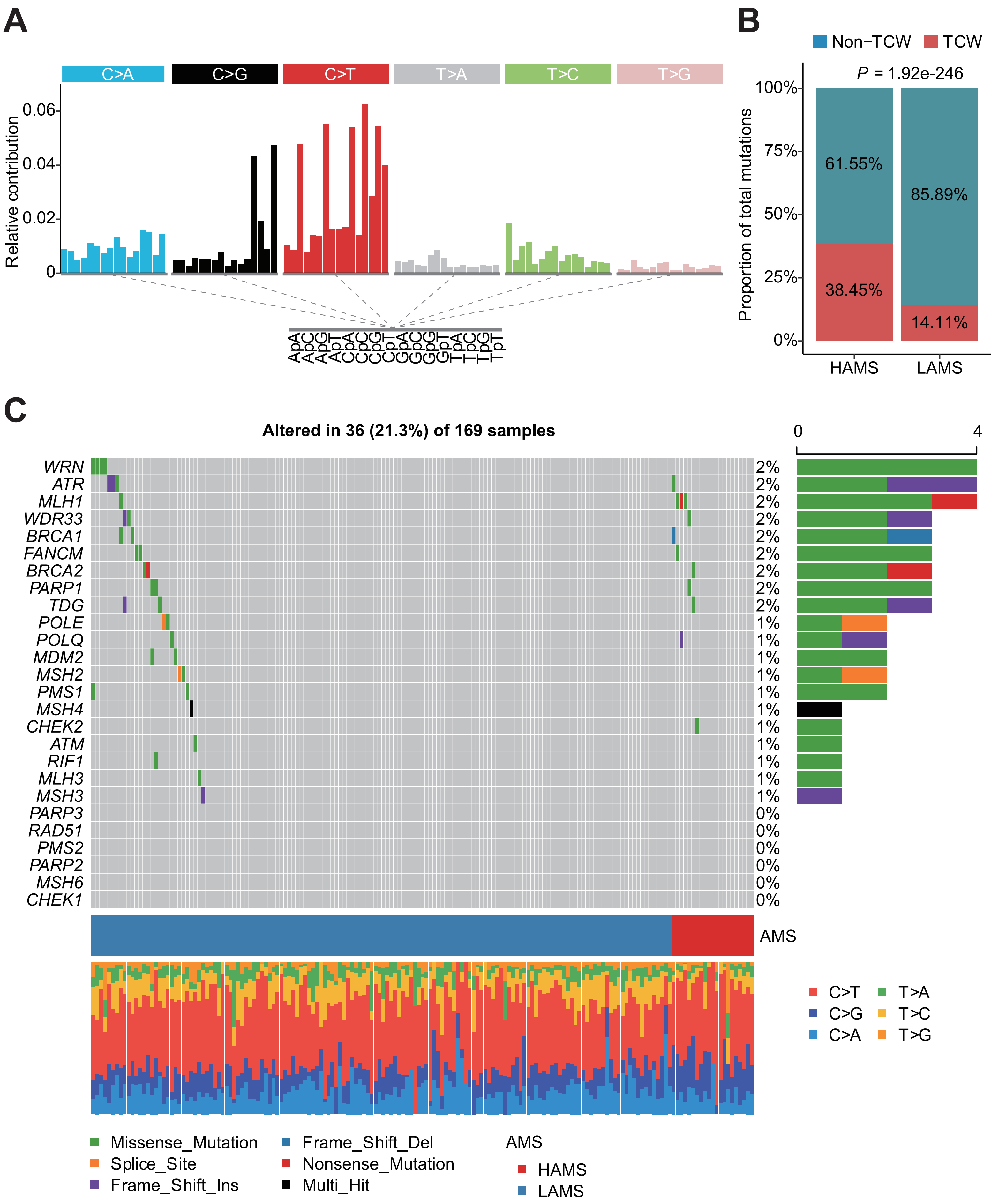

Yang et al. Supplementary Figure 2

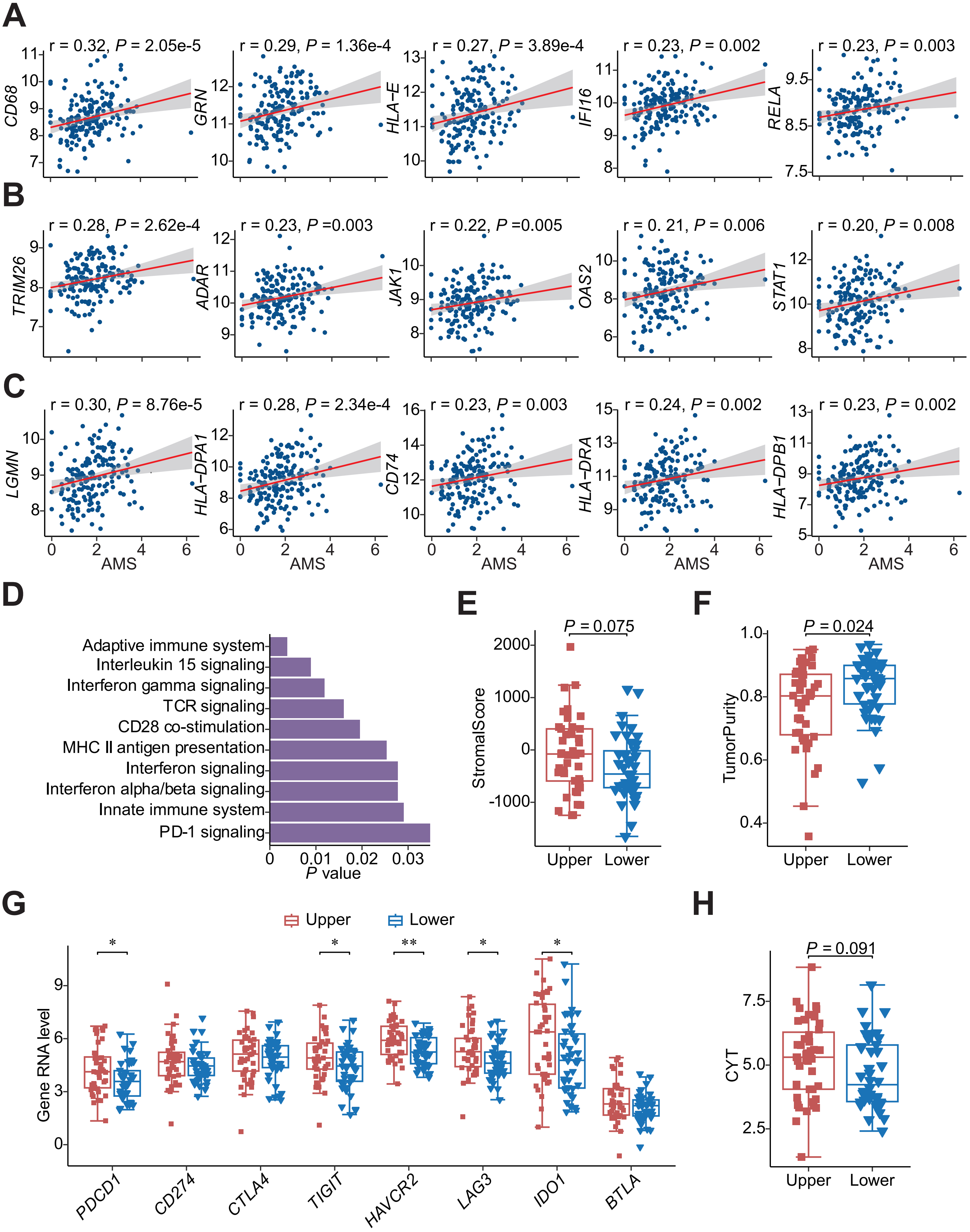

Yang et al. Supplementary Figure 3

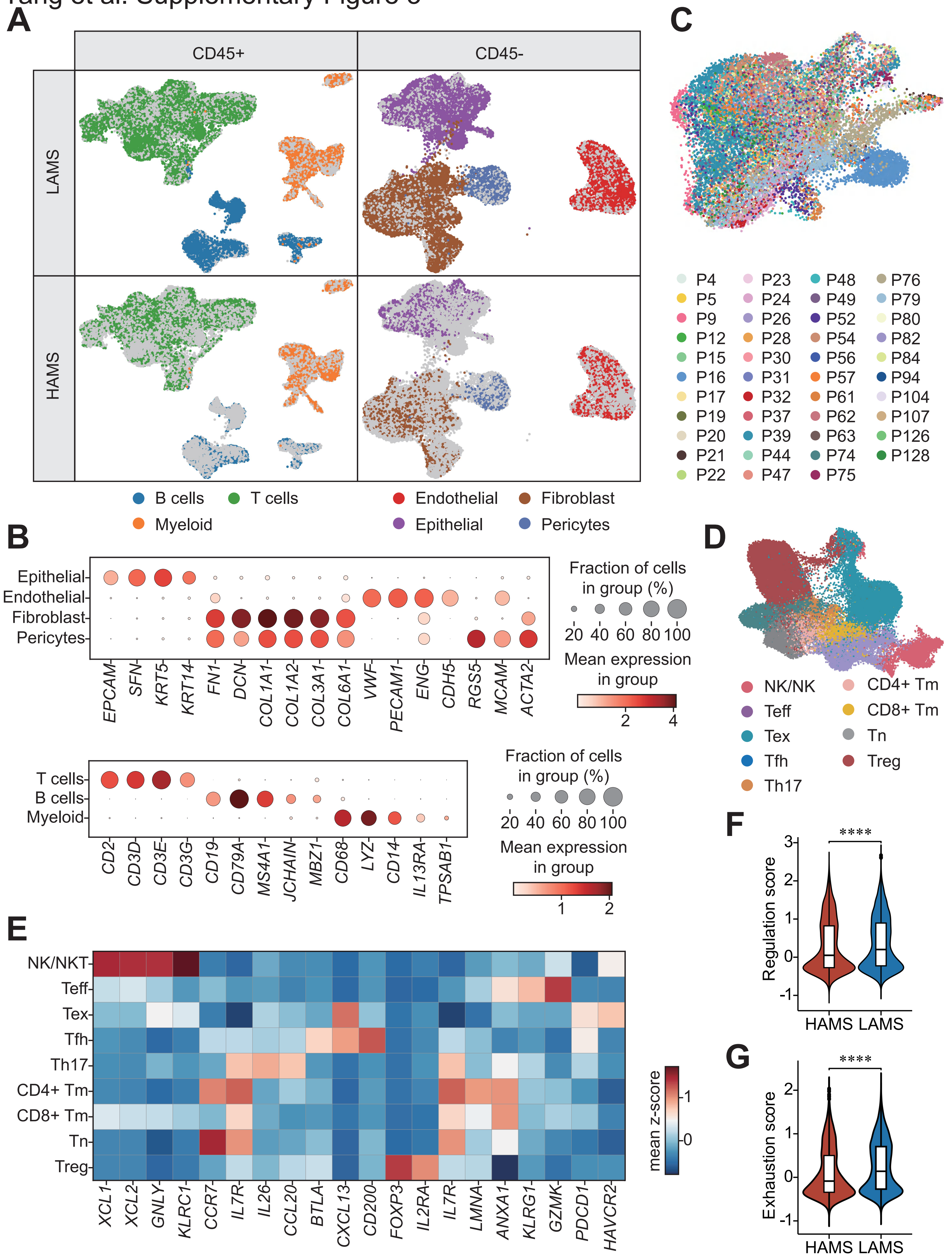

Yang et al. Supplementary Figure 4

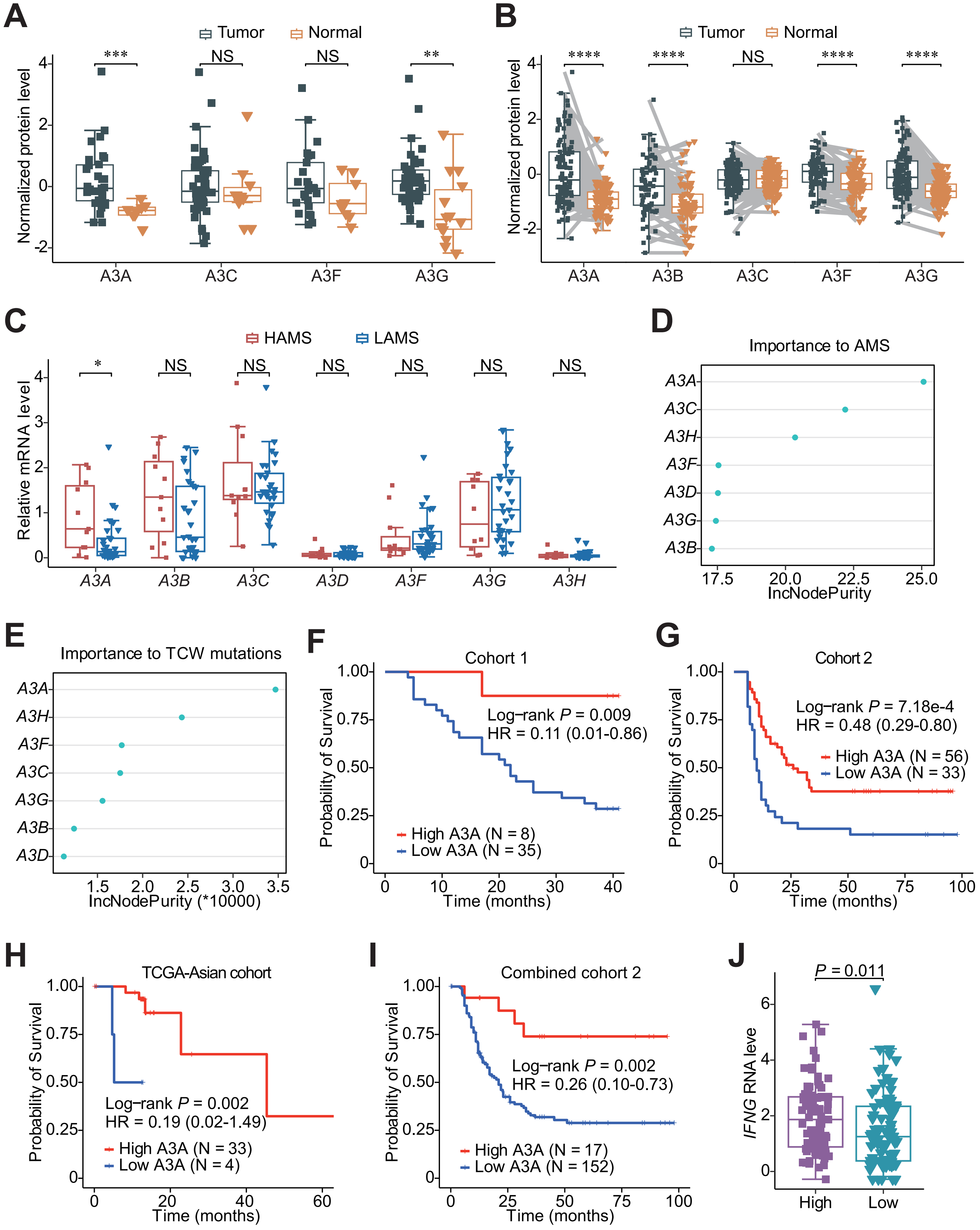

Yang et al. Supplementary Figure 5

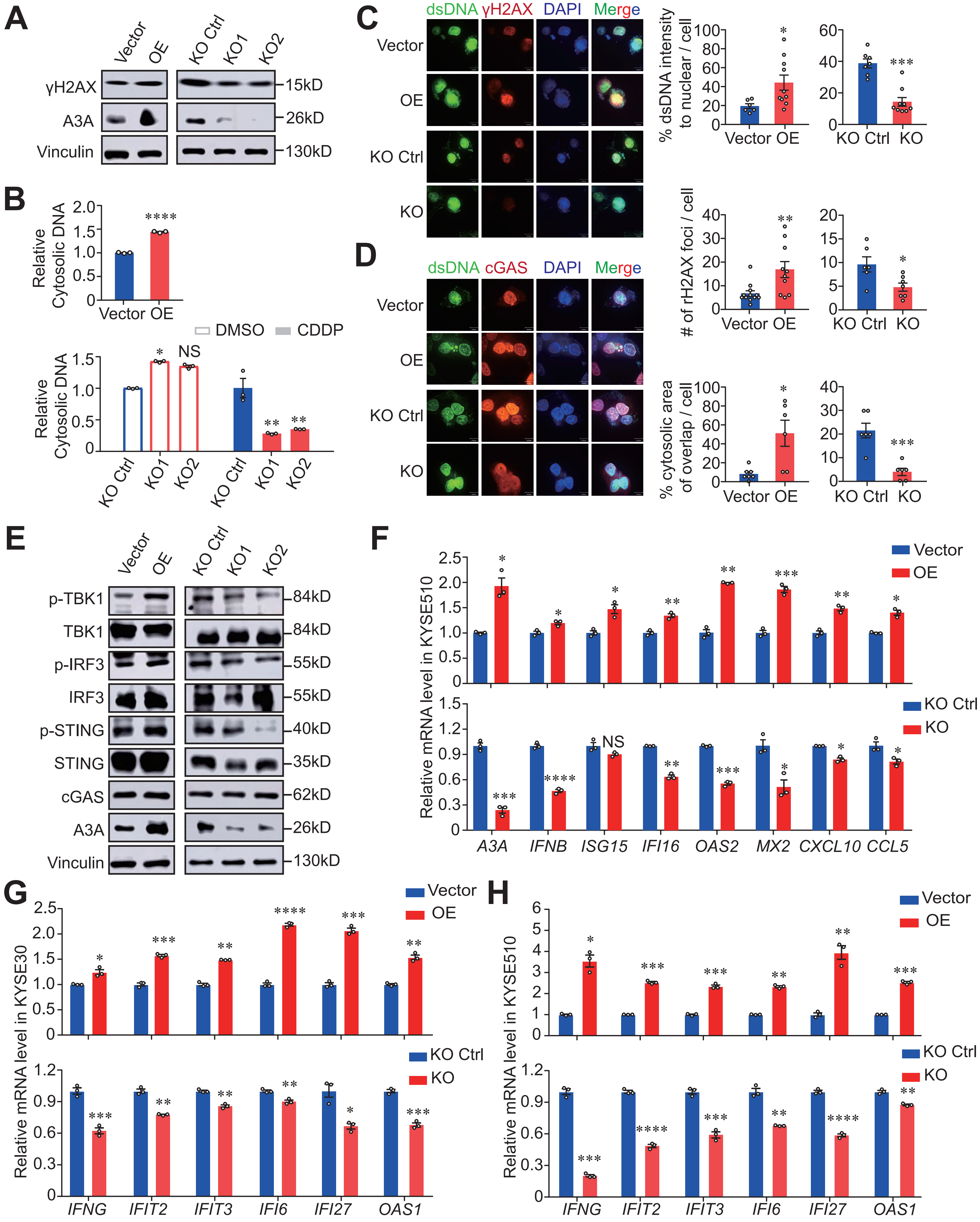

Yang et al. Supplementary Figure 6

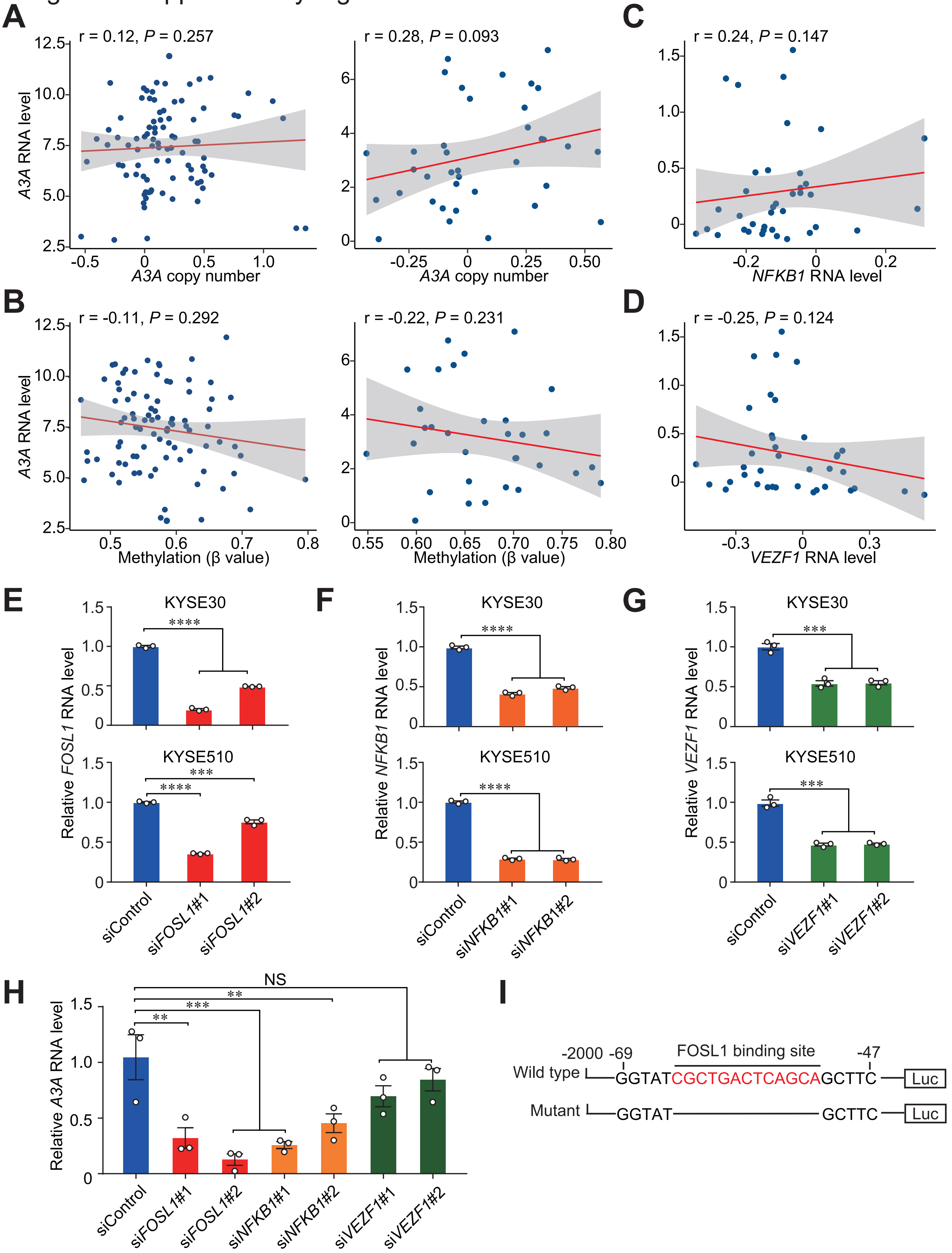

Yang et al. Supplementary Figure 7

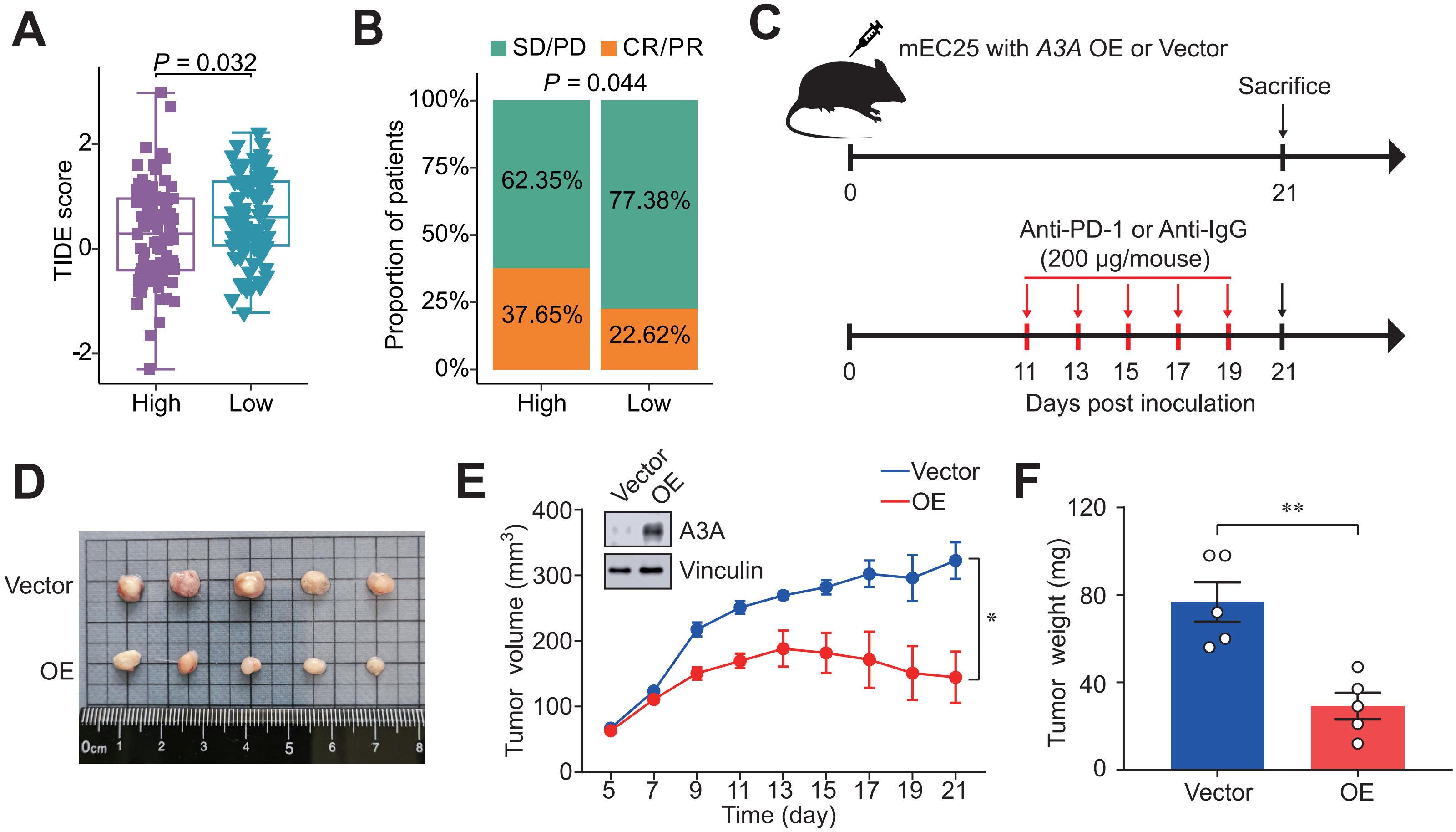

Table S1. Clinical characteristics of patients with ESCC in this study

Sample ID	Data source	Age	Gender	Smoking	Drinking	TNM stage	Survival status	Survival time (month)
P104	Cohort 1	54	Male	Smoker	Drinker	III	Alive	39
P107	Cohort 1	59	Male	Smoker	Drinker	I	Alive	39
P12	Cohort 1	72	Male	Smoker	non-Drinker	II	Alive	41
P126	Cohort 1	57	Male	Smoker	Drinker	III	Alive	37
P128	Cohort 1	62	Male	non-Smoker	non-Drinker	II	Alive	37
P15	Cohort 1	73	Female	non-Smoker	non-Drinker	II	Deceased	9
P16	Cohort 1	70	Male	non-Smoker	non-Drinker	I	Deceased	4
P17	Cohort 1	75	Female	non-Smoker	non-Drinker	I	Alive	41
P19	Cohort 1	40	Female	non-Smoker	non-Drinker	II	Alive	41
P20	Cohort 1	70	Male	Smoker	Drinker	III	Alive	41
P21	Cohort 1	64	Male	non-Smoker	non-Drinker	III	Deceased	17
P22	Cohort 1	60	Male	Smoker	Drinker	II	Deceased	37
P23	Cohort 1	63	Male	Smoker	Drinker	III	Deceased	23
P24	Cohort 1	54	Male	Smoker	non-Drinker	III	Deceased	12
P26	Cohort 1	78	Male	non-Smoker	non-Drinker	III	Deceased	26
P28	Cohort 1	64	Male	Smoker	Drinker	III	Deceased	12
P30	Cohort 1	65	Female	non-Smoker	non-Drinker	I	Deceased	17
P31	Cohort 1	65	Male	Smoker	non-Drinker	I	Deceased	5
P32	Cohort 1	61	Male	Smoker	non-Drinker	II	Alive	41
P37	Cohort 1	70	Male	non-Smoker	non-Drinker	III	Deceased	22

P39	Cohort 1	61	Male	Smoker	non-Drinker	II	Deceased	10
P4	Cohort 1	70	Female	non-Smoker	non-Drinker	I	Deceased	22
P44	Cohort 1	77	Male	Smoker	non-Drinker	III	Deceased	5
P47	Cohort 1	47	Male	Smoker	Drinker	III	Deceased	13
P48	Cohort 1	55	Female	non-Smoker	non-Drinker	I	Alive	40
P49	Cohort 1	59	Female	non-Smoker	non-Drinker	I	Alive	40
P5	Cohort 1	48	Male	non-Smoker	non-Drinker	III	Alive	41
P52	Cohort 1	58	Male	Smoker	Drinker	I	Deceased	21
P54	Cohort 1	74	Female	non-Smoker	non-Drinker	II	Deceased	7
P56	Cohort 1	56	Male	Smoker	Drinker	I	Deceased	5
P57	Cohort 1	58	Male	Smoker	Drinker	III	Alive	40
P61	Cohort 1	69	Female	non-Smoker	non-Drinker	II	Alive	40
P62	Cohort 1	70	Male	non-Smoker	non-Drinker	III	Deceased	35
P63	Cohort 1	64	Female	non-Smoker	non-Drinker	III	Deceased	5
P74	Cohort 1	74	Male	Smoker	non-Drinker	II	Deceased	20
P75	Cohort 1	55	Male	Smoker	Drinker	III	Deceased	31
P76	Cohort 1	77	Male	Smoker	Drinker	I	Deceased	11
P79	Cohort 1	74	Male	Smoker	non-Drinker	I	Deceased	17
P80	Cohort 1	77	Female	non-Smoker	non-Drinker	III	Deceased	26
P82	Cohort 1	72	Male	Smoker	Drinker	I	Alive	40
P84	Cohort 1	69	Female	non-Smoker	non-Drinker	III	Alive	39
P9	Cohort 1	74	Male	Smoker	Drinker	III	Deceased	17
P94	Cohort 1	69	Male	non-Smoker	non-Drinker	III	Alive	39
ESCC_10	Cohort 2	47	Male	Smoker	Drinker	III	Deceased	7

ESCC_12	Cohort 2	53	Male	Smoker	Drinker	III	Deceased	15
ESCC_125	Cohort 2	63	Male	Smoker	Drinker	III	Alive	98
ESCC_130	Cohort 2	58	Male	Smoker	Drinker	III	Deceased	18
ESCC_131	Cohort 2	50	Male	non-Smoker	non-Drinker	II	Alive	96
ESCC_132	Cohort 2	57	Male	Smoker	Drinker	III	Alive	64
ESCC_134	Cohort 2	59	Male	Smoker	Drinker	III	Deceased	19
ESCC_138	Cohort 2	54	Male	Smoker	Drinker	II	Alive	95
ESCC_140	Cohort 2	77	Male	Smoker	Drinker	III	Deceased	6
ESCC_142	Cohort 2	66	Male	Smoker	Drinker	II	Deceased	11
ESCC_143	Cohort 2	63	Male	Smoker	non-Drinker	III	Alive	94
ESCC_144	Cohort 2	64	Female	non-Smoker	non-Drinker	II	Deceased	32
ESCC_145	Cohort 2	45	Female	non-Smoker	non-Drinker	III	Alive	94
ESCC_149	Cohort 2	62	Male	Smoker	Drinker	III	Deceased	9
ESCC_150	Cohort 2	68	Male	Smoker	Drinker	III	Alive	94
ESCC_152	Cohort 2	62	Male	Smoker	Drinker	II	Deceased	34
ESCC_156	Cohort 2	54	Male	Smoker	Drinker	III	Deceased	14
ESCC_158	Cohort 2	63	Male	Smoker	Drinker	III	Deceased	6
ESCC_16	Cohort 2	55	Male	Smoker	Drinker	III	Deceased	9
ESCC_161	Cohort 2	67	Female	non-Smoker	non-Drinker	III	Alive	92
ESCC_162	Cohort 2	66	Male	Smoker	non-Drinker	II	Deceased	11
ESCC_168	Cohort 2	68	Male	Smoker	Drinker	II	Alive	61
ESCC_169	Cohort 2	68	Male	non-Smoker	non-Drinker	III	Deceased	6
ESCC_170	Cohort 2	60	Male	Smoker	Drinker	II	Alive	58
ESCC_171	Cohort 2	57	Male	Smoker	Drinker	III	Alive	57

ESCC_172	Cohort 2	70	Male	non-Smoker	Drinker	III	Deceased	11
ESCC_173	Cohort 2	62	Female	non-Smoker	non-Drinker	II	Alive	89
ESCC_175	Cohort 2	61	Male	Smoker	Drinker	III	Deceased	6
ESCC_178	Cohort 2	55	Male	Smoker	non-Drinker	III	Deceased	7
ESCC_179	Cohort 2	63	Male	Smoker	Drinker	III	Deceased	51
ESCC_182	Cohort 2	65	Male	Smoker	Drinker	III	Deceased	11
ESCC_185	Cohort 2	59	Male	Smoker	Drinker	III	Deceased	16
ESCC_19	Cohort 2	53	Male	Smoker	Drinker	III	Deceased	10
ESCC_191	Cohort 2	48	Female	non-Smoker	non-Drinker	III	Alive	81
ESCC_196	Cohort 2	63	Female	non-Smoker	non-Drinker	III	Deceased	12
ESCC_198	Cohort 2	52	Male	Smoker	Drinker	II	Deceased	23
ESCC_199	Cohort 2	56	Male	Smoker	Drinker	III	Deceased	13
ESCC_201	Cohort 2	58	Male	Smoker	Drinker	III	Alive	87
ESCC_206	Cohort 2	56	Male	Smoker	Drinker	III	Deceased	9
ESCC_208	Cohort 2	53	Male	Smoker	Drinker	III	Deceased	7
ESCC_21	Cohort 2	55	Male	Smoker	Drinker	III	Deceased	28
ESCC_210	Cohort 2	51	Male	Smoker	Drinker	II	Deceased	22
ESCC_213	Cohort 2	60	Male	Smoker	Drinker	III	Alive	85
ESCC_215	Cohort 2	61	Male	Smoker	Drinker	II	Alive	84
ESCC_220	Cohort 2	58	Male	Smoker	Drinker	III	Deceased	6
ESCC_222	Cohort 2	56	Male	Smoker	Drinker	III	Deceased	26
ESCC_223	Cohort 2	76	Male	non-Smoker	Drinker	III	Deceased	11
ESCC_224	Cohort 2	58	Female	non-Smoker	Drinker	III	Deceased	12
ESCC_225	Cohort 2	67	Male	non-Smoker	Drinker	II	Deceased	6

ESCC_23	Cohort 2	60	Female	non-Smoker	non-Drinker	III	Deceased	33
ESCC_234	Cohort 2	64	Male	Smoker	Drinker	III	Deceased	16
ESCC_239	Cohort 2	62	Male	Smoker	Drinker	III	Deceased	7
ESCC_24	Cohort 2	43	Male	Smoker	Drinker	III	Deceased	8
ESCC_240	Cohort 2	66	Male	Smoker	Drinker	III	Deceased	12
ESCC_243	Cohort 2	45	Male	Smoker	Drinker	III	Deceased	9
ESCC_245	Cohort 2	69	Male	Smoker	Drinker	III	Deceased	9
ESCC_246	Cohort 2	63	Male	Smoker	Drinker	III	Deceased	8
ESCC_249	Cohort 2	64	Male	Smoker	Drinker	III	Deceased	7
ESCC_26	Cohort 2	56	Male	Smoker	Drinker	II	Deceased	6
ESCC_27	Cohort 2	54	Male	Smoker	non-Drinker	III	Deceased	9
ESCC_3	Cohort 2	61	Male	Smoker	Drinker	II	Deceased	12
ESCC_35	Cohort 2	53	Male	Smoker	Drinker	III	Deceased	32
ESCC_36	Cohort 2	65	Male	Smoker	Drinker	II	Deceased	6
ESCC_39	Cohort 2	72	Male	non-Smoker	Drinker	II	Deceased	6
ESCC_42	Cohort 2	62	Male	Smoker	Drinker	III	Deceased	28
ESCC_48	Cohort 2	55	Male	Smoker	Drinker	III	Deceased	23
ESCC_50	Cohort 2	53	Male	Smoker	Drinker	II	Deceased	11
ESCC_54	Cohort 2	44	Male	Smoker	Drinker	III	Deceased	10
ESCC_55	Cohort 2	61	Male	Smoker	non-Drinker	IV	Deceased	21
ESCC_57	Cohort 2	68	Male	Smoker	Drinker	II	Deceased	12
ESCC_58	Cohort 2	47	Male	Smoker	Drinker	III	Deceased	14
ESCC_60	Cohort 2	54	Male	Smoker	Drinker	II	Alive	18
ESCC_61	Cohort 2	58	Male	Smoker	Drinker	III	Deceased	9

ESCC_62	Cohort 2	75	Male	Smoker	Drinker	III	Deceased	33
ESCC_64	Cohort 2	68	Male	Smoker	Drinker	III	Deceased	21
ESCC_65	Cohort 2	54	Male	Smoker	Drinker	III	Deceased	10
ESCC_E11	Cohort 2	56	Female	non-Smoker	non-Drinker	II	Alive	59
ESCC_E25	Cohort 2	72	Female	non-Smoker	non-Drinker	II	Alive	26
ESCC_E26	Cohort 2	49	Male	Smoker	Drinker	II	Alive	58
ESCC_E3	Cohort 2	75	Male	Smoker	non-Drinker	II	Alive	60
ESCC_E30	Cohort 2	54	Male	Smoker	Drinker	III	Deceased	21
ESCC_E34	Cohort 2	64	Male	Smoker	Drinker	III	Alive	58
ESCC_E45	Cohort 2	61	Male	Smoker	Drinker	III	Deceased	14
ESCC_E47	Cohort 2	64	Male	Smoker	Drinker	III	Deceased	12
ESCC_E50	Cohort 2	67	Male	Smoker	Drinker	III	Alive	24
ESCC_E74	Cohort 2	71	Male	Smoker	non-Drinker	II	Alive	53
ESCC_E75	Cohort 2	64	Male	Smoker	non-Drinker	III	Alive	53
ESCC_E78	Cohort 2	66	Male	Smoker	Drinker	III	Alive	52
ESCC_E79	Cohort 2	62	Male	Smoker	Drinker	III	Alive	52
TCGA-VR-A8EW	TCGA-Asian cohort	57	Male	Smoker	Drinker	III	Deceased	8
TCGA-LN-A9FR	TCGA-Asian cohort	70	Male	non-Smoker	Drinker	II	Alive	12
TCGA-LN-A9FQ	TCGA-Asian cohort	62	Male	non-Smoker	Drinker	II	Alive	13
TCGA-LN-A9FP	TCGA-Asian cohort	60	Female	non-Smoker	Drinker	II	Alive	12
TCGA-LN-A9FO	TCGA-Asian cohort	42	Male	Smoker	non-Drinker	II	Alive	0
TCGA-LN-A8I1	TCGA-Asian cohort	67	Female	non-Smoker	Drinker	II	Alive	13
TCGA-LN-A8I0	TCGA-Asian cohort	52	Male	Smoker	Drinker	II	Alive	14
TCGA-LN-A8HZ	TCGA-Asian cohort	56	Male	NA	Drinker	II	Alive	13

TCGA-LN-A7HZ	TCGA-Asian cohort	49	Male	Smoker	Drinker	II	Alive	13
TCGA-LN-A7HY	TCGA-Asian cohort	50	Male	non-Smoker	Drinker	III	Alive	12
TCGA-LN-A7HX	TCGA-Asian cohort	72	Male	Smoker	Drinker	II	Alive	12
TCGA-LN-A7HW	TCGA-Asian cohort	59	Male	Smoker	Drinker	II	Alive	12
TCGA-LN-A7HV	TCGA-Asian cohort	58	Male	Smoker	Drinker	II	Alive	11
TCGA-LN-A5U7	TCGA-Asian cohort	46	Male	Smoker	Drinker	II	Alive	26
TCGA-LN-A5U6	TCGA-Asian cohort	54	Male	Smoker	Drinker	II	Alive	13
TCGA-LN-A5U5	TCGA-Asian cohort	57	Male	non-Smoker	Drinker	IV	Deceased	5
TCGA-LN-A4MQ	TCGA-Asian cohort	46	Male	Smoker	non-Drinker	III	Alive	13
TCGA-LN-A4A9	TCGA-Asian cohort	58	Male	Smoker	Drinker	II	Deceased	12
TCGA-LN-A4A8	TCGA-Asian cohort	52	Male	non-Smoker	Drinker	II	Alive	16
TCGA-LN-A4A5	TCGA-Asian cohort	49	Male	non-Smoker	Drinker	II	Deceased	23
TCGA-LN-A4A4	TCGA-Asian cohort	36	Male	non-Smoker	Drinker	III	Alive	13
TCGA-LN-A4A3	TCGA-Asian cohort	61	Male	Smoker	non-Drinker	III	Alive	13
TCGA-LN-A4A1	TCGA-Asian cohort	60	Male	non-Smoker	Drinker	II	Alive	13
TCGA-LN-A49Y	TCGA-Asian cohort	77	Male	non-Smoker	Drinker	II	Alive	13
TCGA-LN-A49X	TCGA-Asian cohort	44	Male	non-Smoker	non-Drinker	II	Alive	13
TCGA-LN-A49W	TCGA-Asian cohort	73	Male	Smoker	Drinker	III	Alive	13
TCGA-LN-A49U	TCGA-Asian cohort	62	Male	Smoker	Drinker	II	Alive	16
TCGA-LN-A49S	TCGA-Asian cohort	59	Male	Smoker	non-Drinker	II	Alive	13
TCGA-LN-A49P	TCGA-Asian cohort	71	Male	Smoker	Drinker	II	Alive	13
TCGA-LN-A49O	TCGA-Asian cohort	47	Male	Smoker	Drinker	II	Alive	14
TCGA-LN-A49M	TCGA-Asian cohort	62	Male	non-Smoker	non-Drinker	II	Alive	13
TCGA-JY-A6FD	TCGA-Asian cohort	51	Female	non-Smoker	Drinker	II	Alive	69

TCGA-JY-A6FA	TCGA-Asian cohort	51	Male	Smoker	Drinker	II	Deceased	45
TCGA-IG-A97H	TCGA-Asian cohort	36	Male	Smoker	Drinker	II	Alive	15
TCGA-IG-A8O2	TCGA-Asian cohort	62	Male	Smoker	Drinker	III	Deceased	5
TCGA-IG-A625	TCGA-Asian cohort	60	Male	non-Smoker	Drinker	III	Deceased	13
TCGA-IG-A50L	TCGA-Asian cohort	58	Male	Smoker	non-Drinker	III	Alive	1

Table S2. Stress marker genes

FOS	*HSPA8*
HSPA1A	*MT1*
JUN	*IER2*
FOSB	*DNAJA1*
JUNB	*SOCS3*
EGR1	*ATF3*
HSPA1B	*JUND*
UBC	*CEBPB*
ZFP36	*ID3*
HSPB1	*PPP1R15A*
HSP90AA1	*HSPE1*
MT2	*CXCL1*
DNAJB1	*DUSP1*
BTG2	*HSP90AB1*
NR4A1	*NFKBIA*
CEBPD	*HSPH1*

Table S3. Genes for functional scores of epithelial and T cells

Antigen presentation score	Exhaustion score	Regulation score
IFI6	*HAVCR2*	*IL2RA*
B2M	*CXCL13*	*FOXP3*
CD74	*KRT86*	*IL1R2*
HLA-B	*PHLDA1*	*TNFRSF4*
HLA-DRA	*GZMB*	*IL32*
HLA-DRB1	*GEM*	*CCR8*
CST3	*ATP8B4*	*AC133644.2*
SAA1	*ACP5*	*TNFRSF18*
C1S	*PLPP1*	*LAIR2*
COL17A1	*KIR2DL4*	*BATF*
CTSB	*LAG3*	*TNFRSF9*
CXCL14	*PRF1*	*LAYN*
CXCL2	*AFAP1L2*	*CTLA4*
GPNMB	*VCAM1*	*AC017002.1*
HLA-DPA1	*RBPJ*	*TNFRSF1B*
HLA-DPB1	*CCL3*	*ZBTB32*
HLA-DRB5	*GOLIM4*	*AC145110.1*
IGFBP2	*GNLY*	*HTATIP2*
IGFBP6	*TIGIT*	*IKZF2*
UBD	*TNFSF4*	*CD177*
BCAM	*GALNT2*	*S100A4*
CXCL10	*CD63*	*CRADD*
CXCL3	*IFITM10*	*IL21R*
DST	*KLRC1*	*DNPH1*
HLA-DQA1	*TNFRSF9*	*SYNGR2*
IL32	*CCL5*	*FANK1*
ISG15	*SRGAP3*	*IL1R1*
LAMC2	*PDLIM4*	*CARD16*
MIA	*FAM166B*	*CD79B*
MT2A	*PRRG4*	*CUL9*
	KLRD1	*CD27*

Table S4. Sequences of siRNAs and primers

	Sequence (5' → 3')
A3A F	GAGAAGGGACAAGCACATGG
A3A R	TGGATCCATCAAGTGTCTGG
A3B F	GACCCTTTGGTCCTTCGAC
A3B R	GCACAGCCCCAGGAGAAG
A3C F	AGCGCTTCAGAAAAGAGTGG
A3C R	AAGTTTCGTTCCGATCGTTG
A3D F	ACCCAAACGTCAGTCGAATC
A3D R	CACATTTCTGCGTGGTTCTC
A3F F	CCGTTTGGACGCAAAGAT
A3F R	CCAGGTGATCTGGAAACACTT
A3G F	CCGAGGACCCGAAGGTTAC
A3G R	TCCAACAGTGCTGAAATTCG
A3H F	AGCTGTGGCCAGAAGCAC
A3H R	CGGAATGTTTCGGCTGTT
ACTIN F	CCAACCGCGAGAAGATGA
ACTIN R	CCAGAGGCGTACAGGGATAG
INFB F	GTCAGAGTGGAAATCCTAAG
INFB R	TATGCAGTACATTAGCCATC
ISG15 F	GAACTCATCTTTGCCAGTA
ISG15 R	ATCTTCTGGGTGATCTGC
IFI16 F	GTTTGCCGCAATGGGTTCC
IFI16 R	ATCTCCATGTTTCGGTCAGCA
IFIT2 F	GACACGGTTAAAGTGTGGAGG
IFIT2 R	TCCAGACGGTAGCTTGCTATT
INFG F	TCGGTAACTGACTTGAATGTCCA
INFG R	TCGCTTCCCTGTTTTAGCTGC
MX2 F	CAGAGGCAGCGGAATCGTAA
MX2 R	TGAAGCTCTAGCTCGGTGTTC
OAS2 F	CTCAGAAGCTGGGTTGGTTTAT
OAS2 R	ACCATCTCGTCGATCAGTGTC
CCL5 F	AGCAGTCGTCTTTGTCAC
CCL5 R	TAGCTCATCTCCAAAGAGTT
CXCL10 F	CTGAGCCTACAGCAGAGGAAC
CXCL10 R	GATGCAGGTACAGCGTACAGT
IFI6 F	GGTCTGCGATCCTGAATGGG
IFI6 R	TCACTATCGAGATACTTGTGGGT
IFI27 F	TGCTCTCACCTCATCAGCAGT
IFI27 R	CACAACTCCTCCAATCACAACT
OAS1 F	AGCTTCGTACTGAGTTCGCTC

OAS1 R	CCAGTCAACTGACCCAGGG
IFIT3 F	AAAAGCCCAACAACCCAGAAT
IFIT3 R	CGTATTGGTTATCAGGACTCAGC
NFKB1 F	GGTGCGGCTCATGTTTACAG
NFKB1 R	GATGGCGTCTGATACCACGG
VEZF1 F	AACCCAGTAAGCCTGTCAAGA
VEZF1 R	ATGGGAGAGCTTGTGTCGATT
FOSL1 F	CAGGCGGAGACTGACAAACTG
FOSL1 R	TCCTTCCGGGATTTTGCAGAT
ChIP-*A3A* F	AGGCATGGCAGAGAACTTCC
ChIP-*A3A* R	TTGCTCAAGGCGTGGTGTTA
FOSL1 siRNA#1	GTACGTCGAAGGCCTTGTGAA
FOSL1 siRNA#2	AGTGGATGGTACAGCCTCATT
NFKB1 siRNA#1	CCAGAGTTTACATCTGATGAT
NFKB1 siRNA#2	CCTTTCCTCTACTATCCTGAA
VEZF1 siRNA#1	GTACTTTGGAACAGTACAAAT
VEZF1 siRNA#2	CCAATACCAATAACTCAGAAA
